# Route of fecal microbiota transplantation delivery determined the dynamics and predictability of donor microbe colonization

**DOI:** 10.1186/s42523-025-00495-9

**Published:** 2025-12-24

**Authors:** Paul Oladele, Wenxuan Dong, Brian T. Richert, Timothy A. Johnson

**Affiliations:** Department of Animal Sciences, Purdue University, 270 S Russell St, Room 2020, West Lafayette, IN USA

## Abstract

**Background:**

Fecal microbiota transplantation (FMT) and the colonization of delivered donor microbes has been reported to improve the negative effects (decrease in body weight, diarrhea, and gut barrier disruption) associated with weaning in pigs. However, delivery of FMT in pigs is still invasive and predicting the colonization or rejection of donor microbes remains challenging. Therefore, this study developed a non-invasive in-feed delivery of FMT and evaluated the effect of FMT mode of delivery on growth performance, gut physiology, microbiota dynamics, and predictability of colonization or rejection of donor microbes in recipient pigs. Forty weaned piglets (10 per group) were administered FMT through one of three routes; oral, rectal, or amended in-feed. The control group was orally administered sterile saline to simulate handling stress.

**Results:**

Pigs in the FMT groups had higher average daily weight gain (ADG) from day 0–2 post-weaning. An increase in community diversity and a shift in the recipient community towards the donor in all FMT groups was observed on day 5. The oral group had the highest colonization (15.12%) and the lowest rejection (19.34%) rates, while colonization was 13.82% and 11.78% in rectal and in-feed group respectively. On day 4, colon crypt depth was increased in all FMT groups but an increase in villus length was only observed in the in-feed group. Colonization and rejection of donor microbes in the recipient animals could be predicted in all routes of administration, but the efficacy of prediction was influenced by the route of delivery. In-feed FMT had the lowest colonization prediction which may have been influenced by the need for voluntary consumption of fecal materials in the in-feed group. The ten most abundant genera (*Prevotella*, *Alloprevotella*, *Phascolarctobacterium*, *Lactobacillus*, *Cloacibacillus*, *Bacteroides*, *Lachnoclostridium*, *Escherichia-Shigella*, unclassified *Lachnospiraceae* sequences, and archaea *Methanobrevibacter*) in the recipient prior to FMT (background community) was the most important feature in predicting colonization for all routes of fecal microbiota transplant.

**Conclusion:**

FMT administered as a lyophilized feed additive shows promise in altering microbiome community structure. While colonization and rejection of donor microbes within the recipient community are predictable, the efficacy of these predictions varies with the route of transplant. This suggests that different prediction models are necessary for each delivery mode of FMT in pigs.

**Supplementary Information:**

The online version contains supplementary material available at 10.1186/s42523-025-00495-9.

## Introduction

The weaning transition is one of the most stressful events in the early life of pigs [1]. An abrupt transition from sow milk to a grain-based solid diet causes a reduction in feed intake, extreme microbiome turnover, and dysbiosis, which can favor the proliferation of intestinal pathogens, such as enterotoxigenic *Escherichia coli* (ETEC) [2]. This often leads to a reduction in growth performance, an increase in the incidence of diarrhea and mortality, which is a significant economic loss to producers [3]. Antimicrobial growth promoters such as prophylactic antibiotics and pharmacological level of zinc oxide have been used to modulate the microbiome and prevent diarrhea in weaning pigs, but the increasing incidence of antibiotic resistant bacteria is a public health concern that has necessitated a need for effective alternatives [2].

The microbiome has emerged as a target for alleviating post-weaning stress [4] due to its important role in immune development and nutrient utilization [2, 5, 6]. Approaches that attempt to modulate the microbiome, such as probiotics, prebiotics, and fecal microbiota transplantation (FMT), have been developed to potentially improve post-weaning diarrhea [7–10]. Probiotics are widely used in weaning pigs to promote gut health by excluding pathogens, restoring microbial balance, and modulating immune responses [11, 12]. They can reduce diarrhea [13], restore microbiota after ETEC challenge [14], and enhance barrier function and immunity [15]. These effects often occur through direct and indirect interactions with the gut epithelium.

Fecal microbiota transplantation, the transfer of a whole community of microbes from a fecal sample to a recipient, is the most complex of all the microbiome-based therapies, because it contains live and dead microbes, dietary remnants, viruses, and microbial metabolites [16]. Recent evidence has shown FMT as an effective strategy for preventing post-weaning diarrhea in pigs [17–19]. However, delivery of FMT to pigs is primarily through oral and rectal delivery, which is invasive, stressful to both pigs and their handlers, and cannot be conducted under commercial high-throughput farm environment because of the large number of animals involved. Therefore, there is a need to develop an improved method of delivering FMT to pigs that is less invasive but has similar effectiveness to the conventional mode of delivery.

Despite reports that FMT can improve animal phenotypes, such as body weight gain, feed efficiency, and disease resilience [17, 20, 21], the efficacy of FMT has not been consistent [2, 22], and the accurate prediction of expected post-intervention microbiome phenotypes is still challenging. While the primary objective of FMT is the reconstitution of the microbiome through the colonization of beneficial microbes from the donor or the displacement of harmful microbes in the recipient [23], this reconstitution can lead to secondary effects, such as recovery of microbial compositional balance [24], restoration of both host and microbial metabolic functions [25, 26], and modulation of the host immune system [27]. The ability to accurately predict colonization or rejection of donor microbes based on the baseline microbial community in the recipient may significantly improve the selection of donor communities and the efficacy of FMT colonization in pigs.

To improve FMT administration in pigs, we developed a method of delivering FMT in the feed of pigs (in-feed FMT) through anaerobic preprocessing and lyophilization of the fecal materials. Lyophilization of human fecal material for FMT has previously been shown to preserve bacterial viability [28, 29]. In human patients, freeze-dried FMT delivered in capsule showed comparable clinical efficacy and engraftment to FMT administered via colonoscopy after two weeks [30, 31] Similarly, rectal enema demonstrated equal or superior clinical efficacy and colonization compared to colonoscopic delivery [32, 33]. In pigs, lyophilized FMT capsules and rectal enema ameliorated diarrhea and colitis. Rectal administration of FMT achieved high colonization efficiency, as it bypasses the acidic gastric environment and delivers the microbial community directly to the hindgut, the primary site of microbial fermentation, thereby minimizing the loss of viable organisms in the stomach [34]. Evidence from prebiotic studies suggests that the feed matrix can provide a buffering effect that protects viable bacteria, enhancing their survival during gastrointestinal transit [35, 36]. We hypothesize that the feed matrix would confer a similar protective effect for in-feed delivery, enabling colonization comparable to oral and rectal delivery and that in-feed FMT would not have a negative effect on the development of the gastrointestinal tract in pigs. Delivery route of FMT has been suggested to affect transplant efficacy in humans, possibly due to transit of inoculum through the low gastric pH of the stomach [37]. Similarly, route of FMT administration may impact the prediction of FMT colonization in animals. Accordingly, we further hypothesize that different delivery modes will affect the prediction of FMT response in pigs. To test these hypotheses, we delivered FMT through oral, rectal, and in-feed administration at weaning and characterized the microbiota structure and gastrointestinal tract development of weaned piglets. We then predicted the post-FMT outcome from pre-FMT microbiota features.

## Results

### Fecal microbiota transplantation improved pig performance and gastrointestinal tract development immediately after weaning

**Fig. 1:**
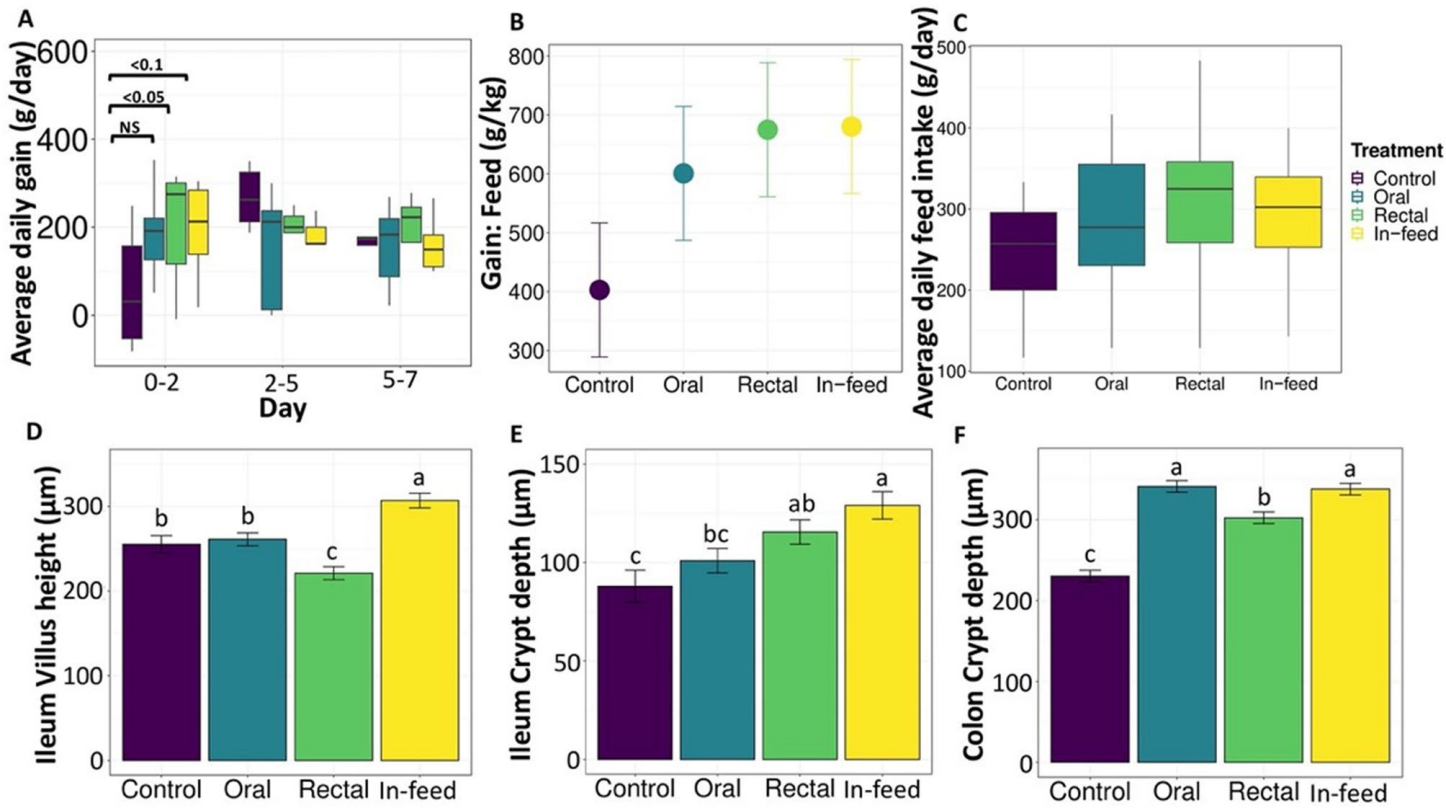
Effect of FMT route of delivery on growth performance and gut morphology in pigs. Effect of FMT on (**A**) Average daily weight gain (ADG), (**B**) Gain to feed ratio (day 0–7), (**C**) Average daily feed intake (ADFI) (day 0–7), (**D**) Ileum villi height on day 4, (**E**) Ileum crypt depth on day 4, and (**F**) Colon crypt depth on day 4. Significant differences within a time point are indicated by p-value, superscript or NS (not significant). For time points or panels with no p-values or superscripts included indicates that no significant differences were identified

FMT increased average daily body weight gain (ADG) from weaning to two days after weaning. The pigs in the rectal group had increased ADG (*P* < 0.05), while those in the in-feed group tended to be higher compared to the control group (*P* = 0.08), but there was no difference on days 5 and 7 (Fig. 1a). There was also no difference in gain: feed (Fig. 1b), average daily feed intake (Fig. 1c), body weight (*P* = 0.75), or diarrhea incidence (*P* = 0.50) in all FMT groups compared to the control group (Fig. S1a-b).

**Fig. 2:**
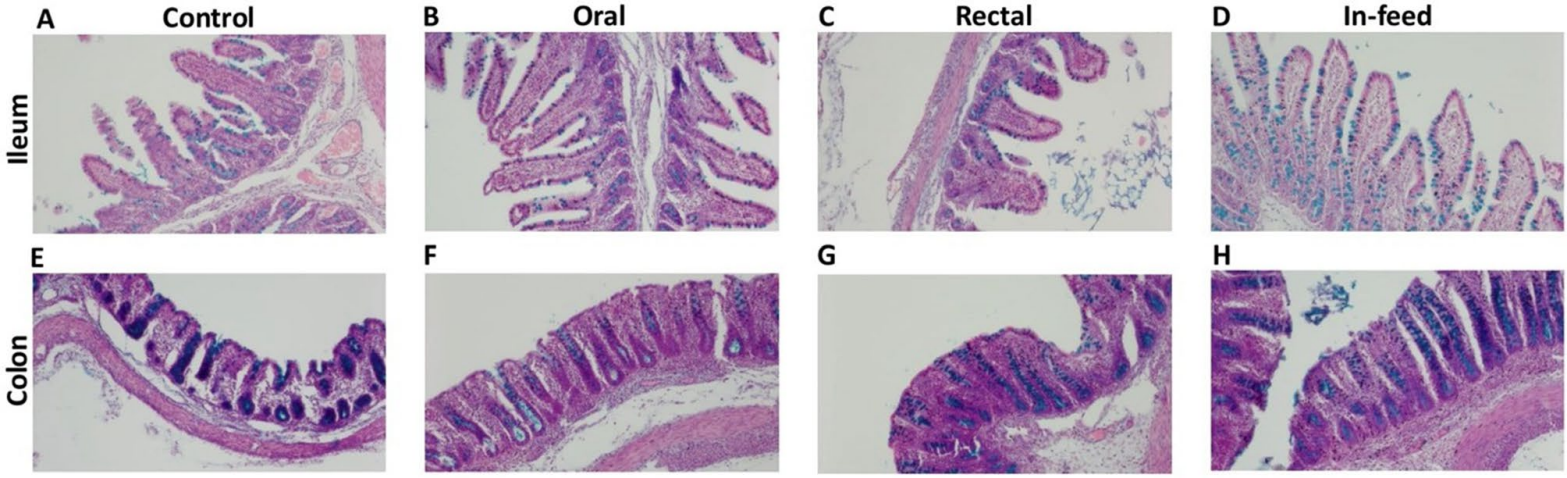
Effects of FMT route of delivery on gut morphology of pigs on day 4. Representative images showing the ileum ((**A**) Control; (**B**) Oral; (**C**) Rectal; (**D**) In-feed) and colon ((**E**) Control; (**F**) Oral; (**G**) Rectal; (**H**) In-feed) segments stained with Alcian blue and Nuclear fast red. Magnification: 10×

To determine if the increased performance immediately after weaning was associated with improvement in gastrointestinal tract development, we measured histomorphology of the pigs (Table S2, Fig. 2). In the ileum of the pigs on day 4, the in-feed FMT group had longer villus length and greater villus perimeter (*P* < 0.001) compared to control, oral, and rectal FMT pigs (Fig. 1d). Also on day 4, crypt depth and crypt perimeter (*P* < 0.001) were increased in the in-feed and rectal FMT groups compared to control pigs (Fig. 1e), while the villus length to crypt depth ratio was lower in the rectal group compared to control and oral treatment groups (Table S2). In the colon on day 4, crypt depth and crypt perimeter (*P* < 0.001) were reduced in the control compared to all FMT groups (Fig. 1f, Table S2). In the ileum on day 8, the oral FMT group had increased villus height, villus perimeter, and crypt perimeter (*P* < 0.001) among all FMT groups, while the rectal group had the highest villus height to crypt depth ratio (*P* < 0.001) among all FMT groups (Table S2). In the colon on day 8, crypt depth and crypt perimeter were reduced in the oral and rectal FMT groups compared to control group (Table S2).

### Fecal microbiota transplantation shifts pig gut microbiota towards the donor

**Fig. 3:**
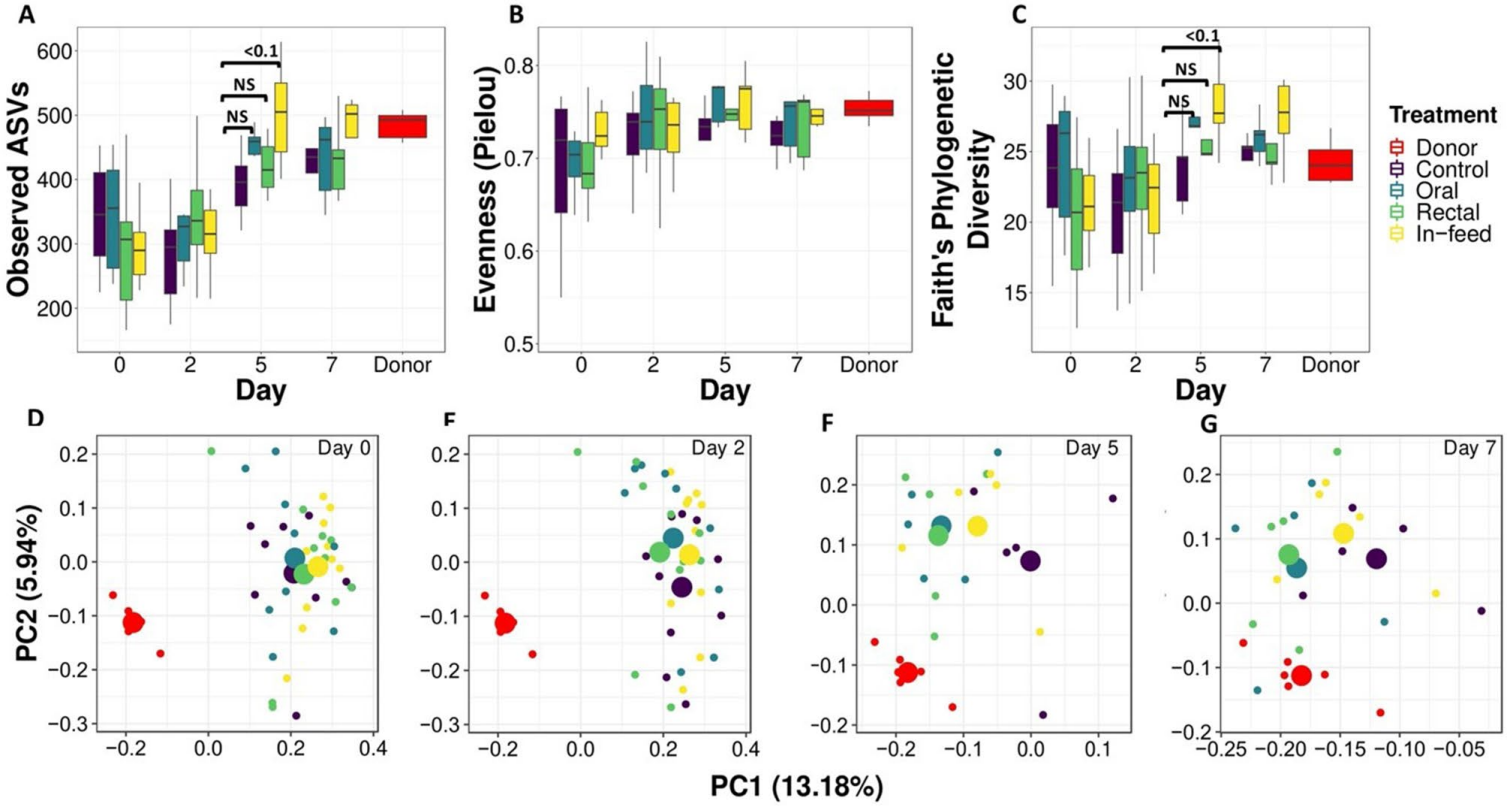
Effects of FMT route of delivery on fecal microbiota dynamics. (**A**) Number of Observed ASVs; (**B**) Pielou’s Evenness; (**C**) Faith’s phylogenetic diversity. Jaccard similarity index on (**D**) day 0; (**E**) day 2; (**F**) day 5; and (**G**) day 7. Significant differences within a time point are indicated by p-value, superscript or NS (not significant). For time points or panels with no p-values or superscripts included indicates that no significant differences were identified

The change in microbiota due to FMT was estimated by alpha diversity, beta diversity, and the relative abundance of taxa. For alpha diversity, richness (number of Observed ASVs; Fig. 3a), and phylogenetic diversity (Faith’s metric; Fig. 3c) were both significantly higher in the in-feed group only on day 5 (*P* < 0.05), but there was no difference (*P* > 0.05) in evenness (Pielou; Fig. 3b).

We used the Jaccard similarity index to estimate community similarity, or the transfer of microbes from donor to recipient, because this index relies on the presence or absence of taxa. From the PCoA plot, all FMT groups clustered together but separately from donors at baseline day 0 (PERMANOVA *P* < 0.001, Fig. 3d). There was no effect of FMT on community structure on day 2 after transplantation (Fig. 3e). However, all FMT groups shifted towards the donor on days 5 and 7, but the oral and rectal groups shifted closer to the donor community than the in-feed group (PERMANOVA *P* < 0.001, Fig. 3f and g).

There was no significant difference in alpha diversity metrics of the microbiota in both the colon and cecum digesta between the FMT groups and control on days 4 and 8 (Fig. S2). However, the microbial community structure of the colon based on Jaccard similarity remained different in the FMT groups (oral, rectal and in-feed) compared to the control group on both day 4 and 8 (PERMANOVA *P* < 0.01, Fig. S3c and d), while the FMT groups were different from control in the cecum only on day 4 (PERMANOVA *P* < 0.01, Fig. S3a and b).

### Colonization dynamics was dependent on the route of fecal microbiota transplant

We profiled the ecological outcomes of microbial taxa from the donor in each recipient pig after FMT. We categorized taxa based on their presence in the donor and recipient before and after FMT. Taxa detected in the donor alone at baseline but present in the recipient after FMT were defined as colonizers, while those that were present in the donor alone at baseline but were still absent in the recipient after FMT were defined as rejecters. Taxa present in both the donor and recipient before and after FMT were defined as co-existers, while those present in the recipient alone before and after FMT were defined as persisters. These categories were used to define six different possible ecological outcomes of FMT. The number of taxa in each ecological category were normalized to the total taxa present in each sample (see Methods section on ecological outcomes for a detailed description).

**Fig. 4:**
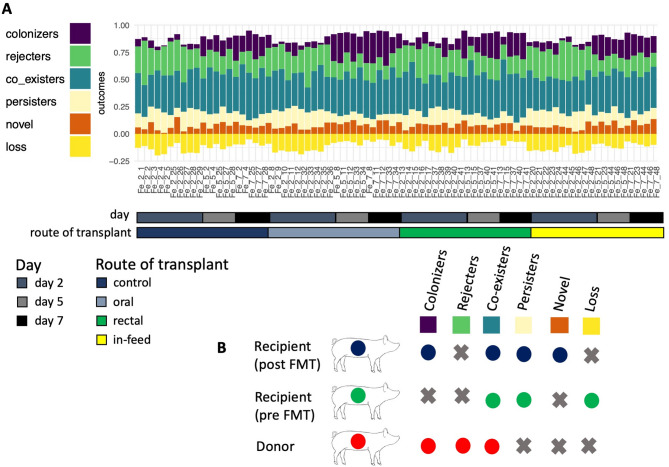
Ecological outcomes of FMT. (**A**) Dynamics of the ecological outcomes of FMT in fecal samples of all treatment groups. (**B**) Illustration of the six ecological outcomes of FMT based on presence or absence of unique taxa in donor and recipient pre-FMT and post-FMT

There was neither complete colonization nor rejection of donor taxa, but colonization and rejection varied among the FMT treatment groups, with the oral group having the highest colonization (15.12%) and the lowest rejection (19.34%) (Fig. 4). Considering all routes of FMT administration, colonization accounted for 13.57%, while rejection was 20.48%. Coexistence post-FMT accounted for most of the taxa in all FMT groups (34.85%), and it was stable alongside persistence (11.51%) all through the duration of the experiment post-FMT. In all FMT groups, colonization increased over time from day 2 to day 7. We observed the presence of some donor taxa in the control group (colonization). The observed colonization of the donor taxa in the control pigs was expected as the recipient pigs are young pigs (< 35 days old), whose microbiomes were expected to develop naturally to be similar to the donor pigs.

We further identified the taxa colonizing in the FMT group. Firmicutes had the highest number of colonizing taxa on all three sampling days. Most of the Firmicutes persisted until day 7, although some taxa disappeared by days 5 and 7. While Bacteroidetes had fewer colonizing taxa, these taxa persisted throughout the sampling period. Three archaea colonized on day 2, but only two persisted until day 5, and none remained by day 7. The family *Lachnospiraceae*, *Prevotellaceae*, *Ruminococcaceae* and *Oscillospiraceae* had the highest number of colonizing taxa in the FMT groups. At the genus level in all FMT groups, *Lachnospiraceae_NK4A136_group*, *Clostridium_sensu_stricto_6*, *Dorea* and *Marvinbryantia* were among the high colonizing genera while *Libanicoccus*, *Senegalimassilia*, *Erysipelotrichaceae_UCG-009*, *Butyrivibrio* and *Shuttleworthia* were some of the most rejected genera across the FMT groups. Most of the colonizing taxa were obligate anaerobes, with a few facultative anaerobes also present and fiber fermenters. A complete list of colonizing and rejected taxa on days 2, 5, and 7 is provided in Tables S3-8.

### Species abundance in recipient before FMT drives colonization and rejection dynamics

**Fig. 5:**
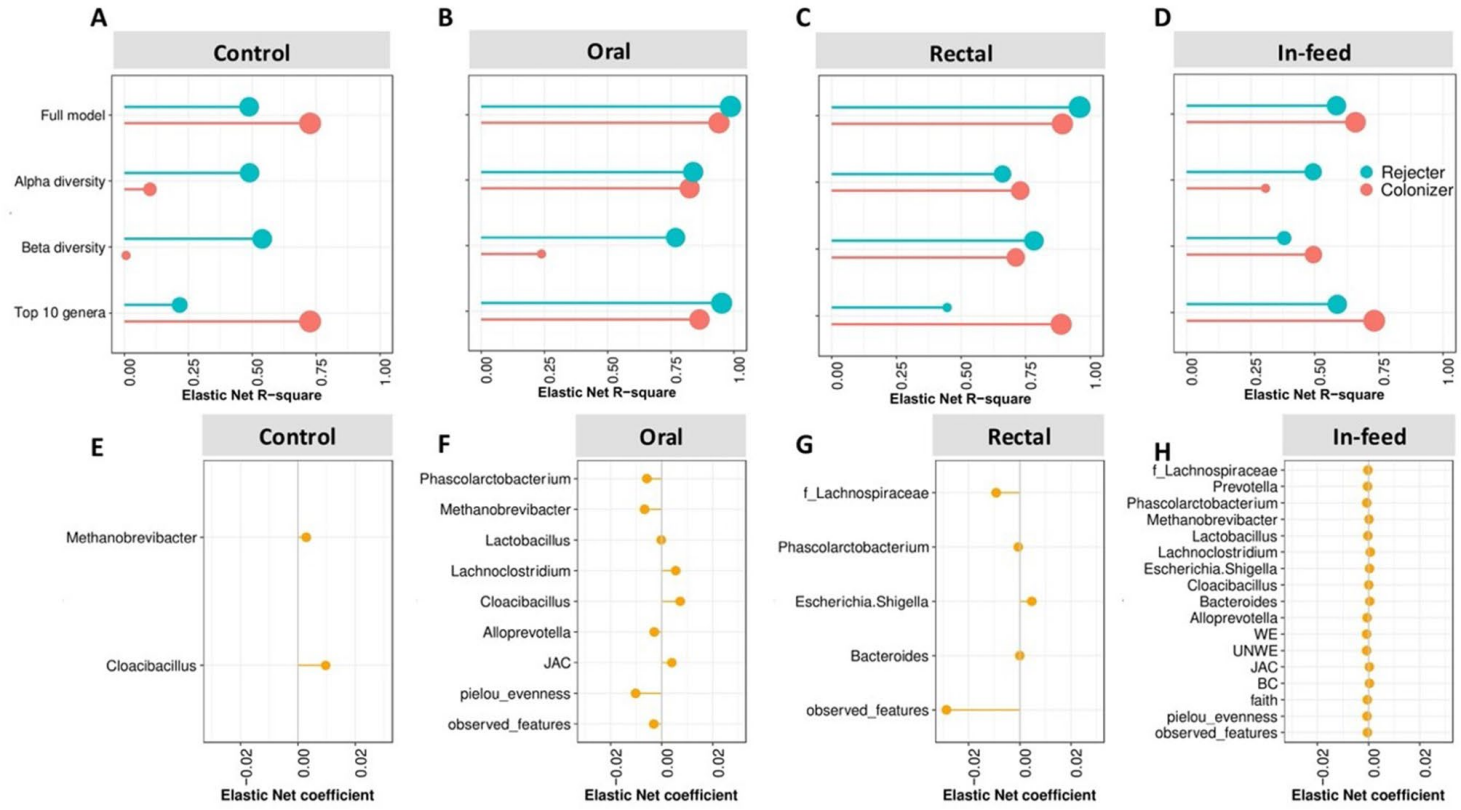
Prediction of the colonization of donor taxa in recipients based on the pre-FMT recipient microbiota features using cross-validated Elastic Net regularized regression. Predictors were divided into alpha diversity (Number of Observed ASVs, Evenness, and Faith), Beta diversity (Bray Curtis, Jaccard, Weighted UniFrac, Unweighted UniFrac), and the ten most abundant taxa and the combination of all categories as the “full model”. Accuracy of prediction of each model is shown as R^2^ for (**A**) Control; (**B**) Oral; (**C**) Rectal; (**D**) In-feed. Variable coefficient and directionality of the full model for (**E**) Control; (**F**) Oral; (**G**) Rectal; (**H**) In-feed

To identify recipient factors that are associated with FMT outcomes, we used a machine learning algorithm trained with an Elastic net regularized regression to predict colonization and rejection. We used microbiome characteristics from each pre-FMT recipient as predictors of post-FMT taxa colonization: alpha diversity, beta diversity, and the ten most abundant taxa in the recipient prior to FMT (see Methods). We then built Elastic net models for each predictor category and the full model using all categories. The accuracy of the model (Fig. 5) varied based on the route of transplantation, but the ten most abundant pre-FMT taxa (R^2^ for oral = 86.37%, rectal = 88.71%, and in-feed = 73.18%) generally explained the highest level of colonization variability by all FMT groups. The full model was required to achieve the highest prediction accuracy of rejection in all FMT groups. In the oral group, *Lachnoclostridium* and *Cloacibacillus* were important features associated with colonization (Fig. 5f) while an increase in number of observed features was negatively associated with colonization in the rectal group (Fig. 5g). In both oral and rectal groups, *Phascolarctobacterium* was associated with rejection. Although, there were many features associated with both colonization and rejection in the in-feed group, their coefficients were small (Fig. 5h and Fig. S4d). This shows that the prediction of both colonization and rejection dynamics differed, depending on the route through which the FMT was administered to pigs.

## Discussion

Fecal microbiota transplantation (FMT) can effectively improve diarrhea and weight loss in pigs, two of the major symptoms of the weaning transition in swine production, by targeting the gut microbiome. FMT can increase microbiome maturation at weaning by providing taxa to the pig microbiome that will become abundant after weaning, with the goal of avoiding early weaning dysbiosis. However, the oral FMT procedure in pigs is invasive and labor intensive. To improve the ease of delivery and reduce stress in pigs due to the FMT procedure, we developed an in-feed FMT delivery method and compared it to the conventional modes of delivery. FMT by rectal and in-feed delivery modes alleviated the weight loss associated with weaning in pigs [38] on day 2 without any compromise to feed intake of the animals. Previous FMT studies have also reported similar increase in animal growth after FMT [18, 39]. However, in the current study, there was no difference in diarrhea incidence after FMT. While several studies have previously similarly reported that FMT had no effect on post-weaning diarrhea incidence in pigs [39–42], other studies have observed a reduction in post-weaning diarrhea incidence after FMT [19, 43–45]. The inconsistency in the effects of FMT on diarrhea in pigs may be influenced by the timing of administration, number of bacterial cells delivered, and baseline donor or recipient microbiota composition [46]. Most previous pig FMT studies have not reported bacterial cell counts delivered, except Qi et al., who delivered 5 × 10^8^ cells [45]. Additionally, FMT timing varied, ranging from pre- to post-weaning phases. Notably, studies that have shown reduced diarrhea used either sows or adult pigs as donor, while those using younger donors (≤12 weeks) reported more inconsistent results. Given the significant differences in microbiota composition between young and adult pigs, further research is needed to determine the optimal donor age and dosage for effective FMT in swine. Although, diarrhea incidence was not consistent between studies, improvement in performance metrics in our study and others further support the notion that decreased incidence of post-weaning diarrhea may not be the only benefit of FMT.

The weaning transition in pigs is also accompanied by structural changes to the intestinal epithelium [47], such as decreased villus height and increased crypt depth in the ileum. These alterations reduce absorptive surface area, leading to reduced nutrient absorption and subsequently decrease in body weight gain [1, 48]. Dietary interventions to maintain gut structure have been used to mitigate the negative effects of weaning on pig growth performance [49]. Fecal microbiota transplantation has been reported to reduce gut structure disruption associated with weaning by increasing villus height in the ileum [45] and preserving rapid expansion of colonic crypt [19]. In our study, the mode of FMT delivery influenced gut structure. Only in-feed delivery significantly increased ileal villus length while all FMT delivery routes increased both ileal and colonic crypt depths. The increase in ileal villus height is beneficial for pigs, but the physiological implications of increased crypt depth, especially in the colon, during weaning are not yet clear. The increased crypt depth may be due to increased cell renewal in all FMT groups [50], or it could also be related to the role of the colon in water and electrolyte reabsorption and microbial fermentation. Further research is needed to evaluate the effect of weaning and the interaction of dietary and microbial interventions on colonic structure of pigs. Altogether, our results suggest that the mode of delivery may play a role in stimulating the intestinal epithelium.

Response to FMT on the structure of the fecal microbiota as shown by alpha diversity and Jaccard Similarity PCoA plot was not observed on day 2 but became observable at day 5 and remained similar to the donor on day 7. Previous studies have reported similar increases in alpha diversity after FMT in pigs [51]. Although growth performance in the in-feed pigs was superior or comparable to the conventional routes of FMT, the fecal microbiome of the in-feed group was more dissimilar from the donor than the conventional FMT delivery routes. The cause of the lesser change in the fecal microbiome in the in-feed group is not clear.

When FMT is administered via oral route, the donor microbiota is exposed to and have the possibility of colonizing the entire gastrointestinal tract. We do not have extensive sampling of the entire gastrointestinal tract, but the effect of FMT on the microbiota community structure was longer lasting in the colon (8 days) than in the cecum (4 days). This larger effect of FMT on the colon community is expected since the colon is the major site of microbial fermentation in the pig [50] and is more similar to the fecal microbiota which was used as the donor.

One goal of FMT is to achieve colonization of donor taxa or the loss of recipient taxa, thereby driving the desired phenotype. Although the oral route had the highest colonization, the dynamics of colonization or rejection of donor taxa, or the loss of recipient taxa were similar across all transplantation routes but varied among individual pigs. This suggest that regardless of the route used to deliver FMT, colonization of donor taxa in recipient pig will occur. Similar to findings in human FMT studies, coexistence of both donor and recipient taxa was the most dominant ecological outcome (overall average: 34.9%) of FMT [52]. In all FMT groups, average colonization rate accounted for 13.6%. We could not find any previous FMT study in pigs to compare this colonization rate. Human studies have reported variable engraftment (colonization) rates ranging from 4% to 70% [52–54], which may be due to different disease indications for FMT, such as *Clostridioides difficile* infection, and chronic inflammatory bowel disease. The relatively low engraftment rate observed in our study could be because antibiotics were not administered to deplete the microbiome of recipient pig prior to FMT. Contrary to observations in humans where Bacteroidetes were the predominant colonizing taxa after FMT [53, 54], Firmicutes were the predominant colonizing taxa after pig FMT. While the fecal community from different hosts may behave similar during FMT, there are still subtle differences to be considered when designing and implementing FMT study in pigs. In our study, the colonizing taxa were primarily anaerobes, which shows that the FMT preparation procedure was effective in maintaining anaerobic conditions for the donor fecal material. Also, *Dorea*,* Lachnospiraceae_NK4A136_group*, and *Clostridium_sensu_stricto_6*, three of the highest colonizing genera have been shown to be associated with reduced incidence of diarrhea in pigs after weaning [55].

The core microbiome concept implies the presence of a stable microbial component essential for host function [56]. However, definitions of “core” taxa vary across studies. Holman et al. defined core genera as those present in ≥ 90% of samples and identified eight [38]; six of these were consistently detected at all time points in our study. Jun et al. defined core taxa as those present in 100% of samples across all pig ages, identifying three genera [57], of which two were also found in our data. Luo et al. described age-associated genera - *Prevotella*, *Megasphaera*, *Treponema*, and *Faecalibacterium*, as being enriched in the grower stage, while *Streptococcus* and *Clostridium* were associated with the finisher stage [58]. All these taxa, although absent during weaning, successfully colonized the recipients post-FMT, suggesting effective transfer of these core and age-associated taxa from donor to recipient.

Predictability of post-FMT outcomes is important for developing strategies to improve precision FMT for specific indications [59]. Colonization and rejection of donor taxa was predictable by the pre-FMT recipient characteristics (chief among them was the most abundant recipient taxa), but the accuracy of the prediction varied based on the route of FMT administration. This supports previous observations that the route of FMT delivery affects the predictability post-FMT outcomes [54] and that the abundance of taxa in either the recipient alone [52] or both donor and recipient [54] are the most important factors in predicting post-FMT-outcomes rather than community level characteristics such as α-diversity and β-diversity of both donor and recipient.

The ten most abundant taxa, used for predicting FMT outcomes, display ecological traits that enhance their colonization and persistence. For instance, genera from the *Lachnospiraceae* family form endospores [60], enabling survival in harsh environments and successful re-establishment post-transplantation. Another genus, *Prevotella* contributes to gut homeostasis through biofilm formation and fermentation of diverse dietary fibers into short-chain fatty acids [61]. *Phascolarctobacterium* supports microbial cross-feeding by converting succinate into SCFAs [62] while *Methanobrevibacter*, a representative archaeon, expresses mucosa-mimicking surface glycans and adhesin-like proteins that promote epithelial attachment [63]. *Methanobrevibacter* is also able to utilize various fermentation products suggesting its syntrophic role in the gut [63]. *Lactobacillus* species are acid- and osmotolerant and proliferate rapidly in nutrient-rich conditions [64]. *Bacteroides* exhibit metabolic versatility and antagonistic capabilities, notably through their type VI secretion system, which deliver antimicrobial effectors [65]. These taxa exhibit functional traits, including spore formation, biofilm production, cross-feeding, mucosal adhesion, and microbial antagonism, that likely underpin their ecological relevance for predicting FMT outcomes and optimizing donor-recipient matching to modulate gut microbial composition effectively.

In-feed fecal microbiota transplantation (FMT) offers a scalable and cost-effective strategy for FMT delivery in industrial swine production by integrating microbiota directly into feed, eliminating the labor and variability of oral delivery for individual pigs. However, its success hinges on robust biosafety protocols, including comprehensive donor screening for pathogens and standardized microbial profiling which could impact cost. A major challenge to large-scale use of in-feed FMT would be maintaining microbial viability during feed processing (which often involves high heat) and extended storage; a solution would require innovations, encapsulation or post-pelleting application. Additionally, regulatory clarity on donor selection, microbial composition, and safety standards is essential to enable industry-wide adoption. With these considerations, in-feed-FMT could reduce antibiotic reliance and enhance herd productivity. One limitation of our study is that we could not model the effect of donor factors on the predictability of post-FMT outcomes, because we used a single donor source for all FMT groups. Future studies should evaluate the effect of different donors on the engraftment and clinical success of FMT.

In this study, we developed a method of delivering fecal microbiota transplantation in the feed of pigs and compared it to conventional delivery methods. Although the oral route resulted in the highest proportion of taxa colonizing the recipient pig from the donor, the overall colonization pattern was similar across all delivery routes. This confirms our first hypothesis that the in-feed route of FMT would achieve similar colonization rates to conventional oral and rectal FMT delivery methods. Interestingly, FMT improved the average daily weight gain of pigs immediately post-weaning, but this improvement preceded changes in microbiome structure. This suggests that changes in the microbiome may not be the sole indicator of FMT success in pigs. Although all routes of FMT delivery influenced the microbiome and growth performance similarly, it was the in-feed group that most influenced gut morphology. The predictability of the microbiome, which is crucial for the long-term adoption of FMT, was also affected by the delivery route of FMT. Our study opens new frontiers for the effective utilization of FMT to improve pig health during the critical weaning transition.

## Materials and methods

### Fecal microbiota transplantation (FMT)

Fresh voided fecal samples were collected from four healthy, 12-week-old growing pigs and transported on ice to the lab. The fecal samples were screened (by qPCR) for ETEC F4 and F18 [66], and for parasites (nematodes, their eggs, and oocysts) using a fecal floatation test [67]. All samples tested negative for both, and the four fecal samples were then pooled and homogenized by using a blender (Mainstays, Walmart, Bentonville, AR, USA). The homogenized samples were diluted 1:1 in sterile 5% mannitol cryoprotectant (Fisher Scientific, Fair Lawn, NJ, USA). Sterile jars (20.3 × 20.3 × 23.6 cm) (Ball Corporation, Danville, IN, USA) were filled with 200 mL of the diluted samples and frozen at -20 °C before lyophilization with the Labconco FreeZone Bulk Tray dryer (Marshall Scientific, Hampton, NH, USA). Lyophilization was done at -84 °C. The freeze-dried fecal products were stored at 4 °C until used.

Forty pigs, blocked by body weight, were randomly allotted into one of four treatment groups based on the route of delivery of FMT (Control, Oral, Rectal and In-feed, *n* = 10 per treatment). The pigs were housed in individual cages. Five animals from each treatment group were housed in each of two rooms. To prevent cross-contamination between groups, animals of the same treatment group were housed in adjacent cages, and an empty cage was left between groups. Feces were flushed with water from the feces collection tray under the cages each morning for the duration of the experiment. Each pig in all three FMT groups was administered 2.023 × 10^8^ viable cells daily for the first 5 days after weaning. The number of viable cells was determined with Quantom viable staining kits (Logos Biosystems, Anandale, VA, USA) following the manufacturer’s instructions. For both oral and rectal FMT, 0.1 g freeze-dried fecal materials were rehydrated in 2 mL of phosphate-buffered saline (PBS; VWR International, Radnor, PA, USA). For the in-feed group, each morning, any residual feed was removed and freeze-dried fecal material (0.1 g) was mixed into 50 g of animal diet. After this amended feed allotment was consumed, piglets were allowed to eat the standard diet ad libitum. The pigs in the oral and rectal groups were administered microbiota via oral or rectal, respectively, with Prima Bottle Mount Vaccinator (Neogen, Lansing, MI, USA). For both oral and rectal delivery, the metal needle of the vaccinator was replaced with flexible polyethylene tubing (Sioux Chief, Kansas City, MO, USA). Pigs in both the control group, which were not administered FMT, and the in-feed group received an oral drench of PBS to mimic the stress of oral or rectal FMT. All pigs received a 2 mL of liquid, either FMT or PBS, per pig.

### Animal treatment and sample collection

This experiment was approved by the Purdue Animal Care Use Committee (PACUC), and the experiment was carried out according to the approved protocol (protocol #: 2206002276). Forty weaned pigs (Duroc × (Landrace × Large White) barrows) with an average weight of 5.54 ± 0.27 kg were housed individually at the Purdue University Animal Science Research and Education Center (ASREC). On average, the piglets used in the study were weaned 19.1 ± 0.8 days after birth and fed a standard weaning diet without antibiotics (see Table S1 for diet composition). Pigs had continual ad libitum access to feed and water except those in the in-feed group, which were initially given 50 g of the amended feed (which contained 0.1 g of fecal material) each morning, before being given ad libitum access to feed and water for the rest of the day during the FMT treatment period.

**Fig. 6:**
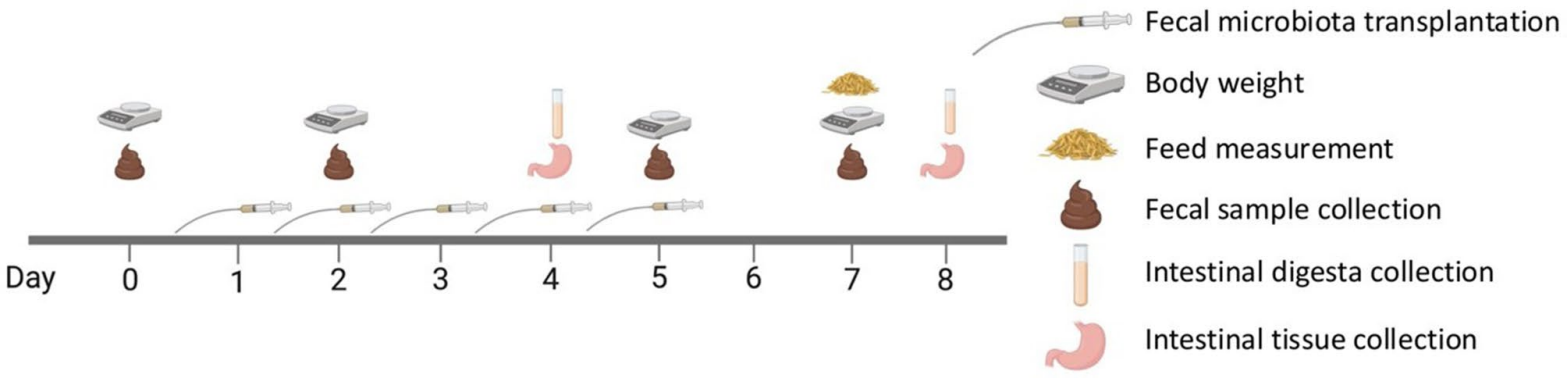
Schematics of experimental procedure. Forty pigs were divided into 4 treatments based on the route of FMT delivery. FMT was performed from day 1–5. Fecal sample and body weight was measured on day 0, 2, 5, and 7. Five animals were slaughtered on days 4 and 8, from which digesta and intestinal tissue was collected. Feed intake for day 0–7 was measured on day 7 and the experiment lasted for 8 days

Animals were weighed and diarrhea scores were recorded on days 0, 2, 5 and 7. Fecal samples were collected through rectal stimulation with a rectal swab on days 0, 2, 5 and 7, while feed intake was measured on day 7 (Fig. 6). Fecal samples were transported on ice to the lab before being stored in a freezer at -20 °C. A diarrhea scoring system ranging from 1 to 5 was used (1 = normal feces, 2 = moist feces, 3 = mild diarrhea, 4 = severe diarrhea, 5 = watery diarrhea) [68]. Five animals per treatment were humanely euthanized on days 4 and 8 through asphyxiation with CO_2_ followed by exsanguination. Digesta from cecum and colon and tissue samples from ileum and colon were collected. Tissue samples were flushed with PBS before being fixed in 10% formalin (Sigma-Aldrich, St. Louis, MO, USA).

### Histology analysis

The terminal ileum and proximal colon of three animals per treatment at each time point were fixed with 10% neutral buffered formalin for 24 h before being transferred to 70% ethanol. Two cross sections were obtained per tissue. The cross sections of fixed tissues were then paraffin embedded at the Purdue University Histology Research Laboratory. Paraffin-embedded tissue blocks were then cut at a thickness of 10 μm and mounted on double-frosted slides (Thermo Fisher Scientific, Frederick, MD, USA). The slides were baked at 60 °C for 20 min, deparaffinized in xylene for 10 min, and rehydrated in a series of solutions with an increasing ratio of distilled water to ethanol. Tissues were then stained with alcian blue (Mercedes Scientific, Lakewood Ranch, FL, USA) and nuclear fast red (G-Biosciences, St. Louis, MO, USA) following standard procedure [69]. Villus height and crypt depth were measured with a microscope with an electronic camera (National Optical and Scientific Instruments, Inc., Schertz, TX, USA) and an ImageJ macro (ImageJ open-source software version 1.8). Villus height and crypt depth were measured for ten intact villi per pig. Villus height was defined as the distance from the tip of the villus to the crypt mouth, whereas crypt depth was defined as the distance from the base of the villus to the muscularis mucosa. Villus height to crypt depth ratio was then calculated.

### DNA extraction and sequencing

Fecal samples were homogenized by stirring, and approximately 0.25 g of representative fecal samples were placed in bead tubes (Qiagen, Germantown, MD). Bead beating was done with TissueLyser II (Qiagen, Germantown, MD). Total DNA was extracted using the PowerFecal Pro DNA kit (Qiagen, Germantown, MD) following the manufacturer’s instructions. The concentration of DNA was quantified with PicoGreen (Thermo Fisher Scientific, Frederick, MD, USA). The V4 region of the 16 S rRNA gene was amplified using 515 F (GTGCCAGCMGCCGCGGTAA) and 806R (GGACTACHVGGGTWTCTAAT) primers, and a pooled amplicon library was prepared following the protocol described by Kozich et al. [70, 71]. A mock community (20-Strain Even Mix 138 Genomic Material; ATCC^®^ MSA-1002™) and PCR-grade water were used as controls during library construction. The amplicon pool was normalized with the SequalPrep Normalization Plate kit (Thermo Fisher Scientific, Frederick, MD, USA). The amplicons were then sequenced with an Illumina MiSeq Sequencer (2 × 250 bp paired end) at the Purdue University Genomics Core Facility.

### Sequence processing

Raw sequences obtained from the 16 S rRNA sequencing were analyzed using Quantitative Insight into Microbial Ecology (QIIME2 v. 2022.8) [72]. The raw sequences from the mock community were compared to the negative control (PCR grade water) to determine the combined error rate due to library preparation and sequencing. The mock community had an error rate of 1.0% and reflected the expected composition. Raw sequences were demultiplexed, and low-quality reads were removed during denoising with DADA2 [73]. During denoising, the forward and reverse sequences were trimmed at position 13, while they were truncated at positions 250 and 222 to obtain sequences with a 50th percentile quality score >35. Forward and reverse reads were then merged and rarefied to a sampling depth of 45,110 reads per sample, which retained all samples and a total of 9,292,660 sequences. Amplicon sequence variants (ASV) were aligned with mafft [74], which was then used to construct a phylogeny with fasttree2 [75]. Alpha diversity metrics were estimated with Observed ASVs as a measure of richness [76], Pielou’s index as a measure of evenness [77], Faith’s index as a measure of phylogenetic diversity [78]. For beta diversity metric, Jaccard similarity metric [79, 80], which considers the presence or absence of ASVs, was used as the primary metric to determine community structure. We consider this metric particularly relevant for studying the establishment of a donor community within the recipient. PCoA was calculated by QIIME2 and visualized with R v. 4.2.2. Bray-Curtis dissimilarity, Unweighted Unifrac and weighted Unifrac were also estimated for use in prediction of colonization and rejection. Taxonomy was assigned to ASVs using the 515 F/806 region of the Silva database (version 138) [81].

### Ecological outcomes of FMT

To assess the microbiota dynamics between each recipient and donor communities after FMT, we defined six possible ecological outcomes of FMT. The rarefied ASV count table collapsed to the genus level was used for determining these outcomes. These outcomes were defined based on the presence or absence of genera in the following sample triad: donor (D), pre-FMT recipient (R), and post-FMT recipient (P). The six outcomes used are illustrated in Fig. 4b and are defined as:

1. Colonization: donor taxa that colonize in the recipient post-FMT $\:\to\:$ the number of unique genera present in both D and P but not R / number of unique genera in P.
2. Rejection: donor taxa that fail to colonize in the recipient post-FMT $\:\to\:$ the number of unique genera present in D but absent in R and P. / number of unique genera in P.
3. Coexistence: donor taxa that are present in the recipient both pre-FMT and post-FMT $\:\to\:$ the number of unique genera present in D, R, and P / number of unique genera in P.
4. Persistence: recipient taxa that are present before and post-FMT $\:\to\:$ the number of unique genera absent in D but present in R and P / number of unique genera in P.
5. Novel: new taxa present in recipients post-FMT $\:\to\:$ the number of unique genera absent in D and R but present in P / number of unique genera in P.
6. Loss: taxa present in the recipient pre-FMT but absent post-FMT $\:\to\:$ the number of unique genera present in R but absent in D and P / number of unique genera in P.

The count of genera in each outcome for each sample was normalized by the number of unique genera present in each sample. Chu et al. [82] used a similar strategy of using the 16 S rRNA gene to categorize taxa in FMT donor and recipients into ecological outcomes. One limitation of this approach is that the 16 S rRNA gene may not differentiate sequences at a strain-level resolution. Because of this limitation, we collapsed the ASVs to the genus level, a level at which it has been documented that the 16 S rRNA gene can reliably identify taxa [83]. We also focused our analysis on the presence or absence of each genus unique to the donor and recipient rather than including relative abundance in each sample when defining FMT outcomes. This method is conservative and could underestimate the FMT colonization obtained. Ecological outcomes for each genus were determined for each individual recipient and then the average of the percent of the community in each outcome was determined for each treatment group.

### Modeling of FMT outcomes

We tested the hypothesis that the efficacy of colonization of an FMT depends on the composition of the resident microbiome of the recipient animal. Because the proportion of coexistence and persistence was similar across time points in all samples, we explored the possibility of predicting two FMT outcomes, colonization, and rejection, in the recipient using baseline characteristics of the recipient microbiome before FMT. We explored 17 predictors based on the main metrics used to investigate the microbial community. These predictors were grouped into three categories: alpha diversity (number of Observed ASVs, Pielou’s evenness, and Faith’s phylogenetic diversity), beta diversity (Bray-Curtis Dissimilarity Index, Jaccard Similarity Index, Unweighted UniFrac Distance and Weighted UniFrac Distance), and the ten most abundant genera of the recipients pre-FMT (*Bacteroides* (6.9%), *Lachnoclostridium* (6.1%), *Prevotella* (4.8%), *Escherichia-Shigella* (4.8%), *Phascolarctobacterium* (4.3%), *Cloacibacillus* (4.3%), archaea *Methanobrevibacter* (3.9%), unclassified *Lachnospiraceae* sequences (3.6%), *Lactobacillus* (3.3%) and *Alloprevotella* (3.3%)) were taken to reduce possible redundancy of low abundant taxa and correlation in relative abundance. Because of the possibility of high multicollinearity between the predictors, the FMT outcomes were modelled using five times five-fold cross-validated Elastic Net regularized regression [84] implemented in the R package glmnet (version 4.1.7). The optimal model was chosen with the cross-validated alpha and lambda values with the smallest Root Mean Square Error (RMSE). FMT outcomes on day two were used for modelling because of the low sample size on days five and seven due to the animals sacrificed on day four.

### Statistical analysis

All analyses and figures were made in R v. 4.2.2. Animal performance data and alpha diversity metrics (Observed ASVs: number of ASVs, Pielou evenness: difference in abundance distribution between ASVs and Faith’s phylogenetic diversity: breadth of taxonomic diversity) except gain: feed were analyzed with a one-way ANOVA to test the difference between treatment groups at each time point. Tukey’s HSD test was used to compare multiple mean when ANOVA test was significant. Gain: Feed was analyzed with Kruskal-Wallis test because the assumptions of parametric test were not satisfied. Permutational multivariate analysis of variance test (PERMANOVA) [85] in QIIME2 was used to test statistical differences in Beta diversity metric (Jaccard Similarity Index) between treatment groups at each time point. Results were considered significant when P *<* 0.05 and trend when 0.05.

$$\:\le\:$$*P* < 0.10.  

## Supplementary Information

Below is the link to the electronic supplementary material.

Supplementary Material 1

## Data Availability

All raw sequencing reads are available in the NCBI sequence read archive (SRA) under BioProject numbers PRJNA1219590 and PRJNA1219559.

## Code availability

All additional files and scripts used in data analysis for this study are available at https://github.com/oluwapaul/fmt1.
