# Supplementary material for: Route of fecal microbiota transplantation delivery determined the dynamics and predictability of donor microbe colonization
Source: Anim Microbiome. 2025 Dec 24;7:130. doi: 10.1186/s42523-025-00495-9 (PMC12729235; doi:10.1186/s42523-025-00495-9)
Supplement: Supplementary file 1 — Supplementary Material 1 [file 42523_2025_495_MOESM1_ESM.docx]

**ADDITIONAL FILE 1**

**Route of fecal microbiota transplantation delivery determined the dynamics and predictability of donor microbe colonization**

Paul Oladele, Wenxuan Dong, Brian Richert, Timothy A. Johnson

Department of Animal Sciences, Purdue University, West Lafayette, Indiana, USA

**Supplemental Tables and Figures**

**Table 1**: Ingredients and nutrient composition of diets, as-fed basis

**Table 2**: Effect of route of FMT on the intestinal morphology of pig

**Table 3**: Colonizing genera in all FMT treatment groups on day 2 post FMT

**Table 4**: Colonizing genera in all FMT treatment groups on day 5 post FMT

**Table 5**: Colonizing genera in all FMT treatment groups on day 7 post FMT

**Table 6**: Rejected genera in all FMT treatment groups on day 2 post FMT

**Table 7:** Rejected genera in all FMT treatment groups on day 5 post FMT

**Table 8:** Rejected genera in all FMT treatment groups on day 7 post FMT

**Fig. S1**: Effect of route of delivery of FMT on body weight (A) and Diarrhea Index (B) of pigs.

**Fig. S2**: Effects of FMT route of delivery on microbiota dynamics cecum and colon digesta. Alpha diversity of cecum digesta represented by (A) Number of Observed ASVs; (B) Pielou’s Evenness; (C) Faith’s phylogenetic diversity. Alpha diversity of colon digesta represented by (D) Number of Observed ASVs; (E) Pielou’s Evenness; (F) Faith’s phylogenetic diversity.

**Fig. S3**: Effects of FMT route of delivery on microbiota dynamics cecum and colon digesta. Beta diversity of cecum digesta represented by Jaccard similarity for cecum on (A) day 4; (B) day 8; and colon on (C) day 4; (D) day 8.

**Fig. S4**: Prediction of the rejection of donor taxa in recipients based on the pre-FMT recipient microbiota features using cross-validated Elastic Net regularized regression. Variable coefficient and directionality of the full model for (A) Control; (B) Oral; (C) Rectal; (D) In-feed.

**Table 1**: Ingredients and nutrient composition of diets, as-fed basis

| Ingredient, % |  |
| --- | --- |
| Corn | 37.265 |
| SBM, 48% CP | 14.150 |
| Soybean oil | 5.000 |
| Limestone | 0.810 |
| MonoCal | 0.300 |
| Vitamin Prx | 0.250 |
| TM Prx | 0.125 |
| Se Prx | 0.050 |
| Phytase | 0.100 |
| Salt | 0.250 |
| Plasma Protein | 5.000 |
| SD Blood Meal | 1.500 |
| Soy Conc. | 5.000 |
| Fish Meal | 4.000 |
| Dried Whey | 25.750 |
| Lysine-HCL | 0.150 |
| DL-Methionine | 0.230 |
| L-Threonine | 0.060 |
| L-Tryptophan | 0.010 |
| Total | 100.000 |
|  |  |
| Calculated Nutrients |  |
| ME, Kcal/kg | 3591.10 |
| SID Lys, % | 1.55 |
| CP,% | 24.43 |
| Ca, % | 0.85 |
| Phos, % | 0.70 |
| Av.P, % | 0.55 |
| Lactose, % | 18.0 |

|  |  |  | Treatment | | | | | SEM | | F-value | | P-value | |
| --- | --- | --- | --- | --- | --- | --- | --- | --- | --- | --- | --- | --- | --- |
|  | Day |  | Control | Oral | Rectal | In-feed |  | |  | |  | |  |
| Ileum | 4 | Villus height, μm | 255.0^b^ | 261.0^b^ | 221.0^c^ | 307.0^a^ | 8.5 | | 15.10 | | < 0.001 | |  |
|  | 4 | Crypt depth, μm | 87.9^c^ | 100.9^bc^ | 115.5^ab^ | 129.0^a^ | 6.9 | | 5.94 | | < 0.001 | |  |
|  | 4 | Villus to Crypt ratio | 3.3^a^ | 2.8^a^ | 2.1^b^ | 2.6^ab^ | 0.2 | | 6.56 | | < 0.001 | |  |
|  | 4 | Villus perimeter | 574.0^b^ | 596.0^b^ | 520.0^b^ | 699.0^a^ | 22.2 | | 20.97 | | < 0.001 | |  |
|  | 4 | Crypt perimeter | 278.0^b^ | 354.0^ab^ | 398.0^a^ | 415.0^a^ | 19.7 | | 7.96 | | < 0.001 | |  |
|  | 8 | Villus length | 259.0^b^ | 314.0^a^ | 265.0^b^ | 224.0^c^ | 7.1 | | 23.78 | | < 0.001 | |  |
|  | 8 | Crypt depth | 102.4^b^ | 154.4^a^ | 93.3^b^ | 138.7^a^ | 7.7 | | 15.02 | | < 0.001 | |  |
|  | 8 | Villus to Crypt ratio | 2.7^ab^ | 2.4^bc^ | 3.1^a^ | 1.7^c^ | 0.2 | | 14.52 | | < 0.001 | |  |
|  | 8 | Villus perimeter | 602.0^b^ | 708.0^a^ | 591.0^b^ | 524.0^c^ | 15.7 | | 20.79 | | < 0.001 | |  |
|  | 8 | Crypt perimeter | 336.0^b^ | 494.0^a^ | 342.0^b^ | 448.0^a^ | 20.1 | | 16.43 | | < 0.001 | |  |
| Colon | 4 | Crypt depth | 230.0^c^ | 341.0^a^ | 302.0^b^ | 338.0^a^ | 7.2 | | 66.73 | | < 0.001 | |  |
|  | 4 | Crypt perimeter | 513.0^c^ | 731.0^a^ | 649.0^b^ | 726.0^a^ | 14.9 | | 58.05 | | < 0.001 | |  |
|  | 8 | Crypt depth | 361.0^a^ | 289.0^c^ | 307.0^bc^ | 332.0^ab^ | 11.5 | | 8.49 | | < 0.001 | |  |
|  | 8 | Crypt perimeter | 777.0^a^ | 624.0^c^ | 663.0^bc^ | 712.0^b^ | 23.1 | | 9.27 | | < 0.001 | |  |

**Table 2**: Effect of route of FMT on the intestinal morphology of pig

Means within a column with different superscripts indicate significant differences (P < 0.05).

**Table 3**: Colonizing genera in all FMT treatment groups on day 2 post FMT

| Taxa | Oral | Rectal | In-feed |
| --- | --- | --- | --- |
| c_Bacilli;o_Erysipelotrichales;f_Erysipelatoclostridiaceae;g_Catenibacterium | 4 | 5 | 2 |
| c_Clostridia;o_Lachnospirales;f_Lachnospiraceae;g_Roseburia | 4 | 2 | 2 |
| c_Clostridia;o_Monoglobales;f_Monoglobaceae;g_Monoglobus | 3 | 2 | 3 |
| c_Clostridia;o_Lachnospirales;f_Lachnospiraceae;g_Oribacterium | 3 | 1 | 4 |
| c_Coriobacteriia;o_Coriobacteriales;f_Atopobiaceae;g_uncultured | 2 | 3 | 2 |
| c_Clostridia;o_Lachnospirales;f_Lachnospiraceae;g_Blautia | 3 | 1 | 2 |
| c_Coriobacteriia;o_Coriobacteriales;f_Coriobacteriaceae;g_Collinsella | 1 | 2 | 3 |
| c_Thermoplasmata;o_Methanomassiliicoccales;f_Methanomethylophilaceae;g_Candidatus_Methanoplasma | 2 | 1 | 2 |
| c_Bacilli;o_Erysipelotrichales;f_Erysipelotrichaceae;g_Erysipelotrichaceae_UCG-006 | 1 | 3 | 1 |
| c_Bacilli;o_RF39;f_RF39;g_RF39 | 1 | 2 | 2 |
| c_Negativicutes;o_Veillonellales-Selenomonadales;f_Veillonellaceae;g_Megasphaera | 1 | 2 | 2 |
| c_Clostridia;o_Lachnospirales;f_Lachnospiraceae;g_Coprococcus | 2 | 1 | 1 |
| c_Clostridia;o_Oscillospirales;f_Ruminococcaceae;g_Faecalibacterium | 2 | 1 | 1 |
| c_Bacteroidia;o_Bacteroidales;f_Prevotellaceae;g_Prevotellaceae_UCG-001 | 1 | 3 | 0 |
| c_Clostridia;o_Lachnospirales;f_Lachnospiraceae;g_[Eubacterium]_hallii_group | 1 | 1 | 2 |
| c_Clostridia;o_Peptostreptococcales-Tissierellales;f_Peptostreptococcaceae;g_Terrisporobacter | 1 | 1 | 2 |
| c_Methanobacteria;o_Methanobacteriales;f_Methanobacteriaceae;g_Methanosphaera | 0 | 4 | 0 |
| c_Clostridia;o_Lachnospirales;f_Lachnospiraceae;g_[Ruminococcus]_gauvreauii_group | 0 | 3 | 1 |
| c_Synergistia;o_Synergistales;f_Synergistaceae;g_Pyramidobacter | 0 | 1 | 3 |
| c_Fibrobacteria;o_Fibrobacterales;f_Fibrobacteraceae;g_Fibrobacter | 3 | 0 | 0 |
| c_Clostridia;o_Oscillospirales;f_Ruminococcaceae;g_Fournierella | 2 | 1 | 0 |
| c_Clostridia;o_Peptostreptococcales-Tissierellales;f_Anaerovoracaceae;_ | 2 | 1 | 0 |
| c_Spirochaetia;o_Spirochaetales;f_Spirochaetaceae;g_Treponema | 2 | 1 | 0 |
| c_Actinobacteria;o_Bifidobacteriales;f_Bifidobacteriaceae;g_Bifidobacterium | 1 | 2 | 0 |
| c_Bacilli;o_Lactobacillales;f_Streptococcaceae;g_Streptococcus | 1 | 2 | 0 |
| c_Clostridia;o_Lachnospirales;f_Lachnospiraceae;g_[Eubacterium]_xylanophilum_group | 1 | 2 | 0 |
| c_Thermoplasmata;o_Methanomassiliicoccales;f_Methanomethylophilaceae;g_uncultured | 1 | 1 | 1 |
| c_Actinobacteria;o_Corynebacteriales;f_Corynebacteriaceae;g_Corynebacterium | 1 | 1 | 1 |
| c_Bacteroidia;o_Bacteroidales;f_Prevotellaceae;g_Prevotellaceae_UCG-004 | 1 | 1 | 1 |
| c_Bacteroidia;o_Bacteroidales;f_Prevotellaceae;g_uncultured | 1 | 1 | 1 |
| c_Bacilli;o_Erysipelotrichales;f_Erysipelotrichaceae;g_Turicibacter | 1 | 1 | 1 |
| c_Negativicutes;o_Veillonellales-Selenomonadales;f_Veillonellaceae;g_Veillonella | 1 | 1 | 1 |
| c_Bacteroidia;o_Bacteroidales;f_Rikenellaceae;g_dgA-11_gut_group | 1 | 0 | 2 |
| c_Clostridia;o_Peptococcales;f_Peptococcaceae;g_uncultured | 1 | 0 | 2 |
| c_Alphaproteobacteria;o_Caulobacterales;f_Caulobacteraceae;g_Brevundimonas | 1 | 0 | 2 |
| c_Negativicutes;o_Veillonellales-Selenomonadales;f_Selenomonadaceae;g_uncultured | 0 | 3 | 0 |
| c_Clostridia;o_Lachnospirales;f_Lachnospiraceae;g_[Ruminococcus]_torques_group | 2 | 0 | 0 |
| c_Clostridia;o_Clostridiales;f_Clostridiaceae;g_Clostridium_sensu_stricto_6 | 1 | 1 | 0 |
| c_Clostridia;o_Oscillospirales;f_UCG-010;g_UCG-010 | 1 | 1 | 0 |
| c_Clostridia;o_Oscillospirales;f_Oscillospiraceae;g_uncultured | 1 | 0 | 1 |
| c_Clostridia;o_Lachnospirales;f_Lachnospiraceae;g_Lachnospiraceae_NK3A20_group | 0 | 2 | 0 |
| c_Clostridia;o_Lachnospirales;f_Lachnospiraceae;g_Lachnospiraceae_UCG-008 | 0 | 2 | 0 |
| c_Clostridia;o_Lachnospirales;f_Lachnospiraceae;g_[Eubacterium]_ruminantium_group | 0 | 2 | 0 |
| c_Clostridia;o_Peptostreptococcales-Tissierellales;f_Peptostreptococcaceae;g_Romboutsia | 0 | 2 | 0 |
| c_Coriobacteriia;o_Coriobacteriales;f_Eggerthellaceae;g_Senegalimassilia | 0 | 1 | 1 |
| c_Coriobacteriia;o_Coriobacteriales;f_uncultured;g_uncultured | 0 | 1 | 1 |
| c_Bacilli;o_Erysipelotrichales;f_Erysipelotrichaceae;g_uncultured | 0 | 1 | 1 |
| c_Clostridia;o_Clostridia_UCG-014;f_Clostridia_UCG-014;g_Clostridia_UCG-014 | 0 | 1 | 1 |
| c_Clostridia;o_Clostridia_vadinBB60_group;f_Clostridia_vadinBB60_group;g_Clostridia_vadinBB60_group | 0 | 1 | 1 |
| c_Clostridia;o_Lachnospirales;f_Lachnospiraceae;g_Lachnospiraceae_NK4A136_group | 0 | 1 | 1 |
| c_Negativicutes;o_Veillonellales-Selenomonadales;f_Selenomonadaceae;g_Mitsuokella | 0 | 1 | 1 |
| c_Clostridia;o_Lachnospirales;f_Lachnospiraceae;g_Lachnospiraceae_AC2044_group | 0 | 0 | 2 |
| c_Negativicutes;o_Veillonellales-Selenomonadales;f_Selenomonadaceae;g_Selenomonas | 1 | 0 | 0 |
| c_Coriobacteriia;o_Coriobacteriales;f_Eggerthellaceae;g_Paraeggerthella | 0 | 1 | 0 |
| c_Clostridia;o_Lachnospirales;f_Lachnospiraceae;g_Lachnospiraceae_UCG-007 | 0 | 1 | 0 |
| c_Clostridia;o_Lachnospirales;f_Lachnospiraceae;g_[Eubacterium]_ventriosum_group | 0 | 1 | 0 |
| c_Clostridia;o_Oscillospirales;f_Butyricicoccaceae;g_UCG-008 | 0 | 1 | 0 |
| c_Clostridia;o_Oscillospirales;f_Ruminococcaceae;g_uncultured | 0 | 1 | 0 |
| c_Clostridia;o_Oscillospirales;f_[Eubacterium]_coprostanoligenes_group;g_coprostanoligenes_group | 0 | 1 | 0 |
| c_Campylobacteria;o_Campylobacterales;f_Campylobacteraceae;g_Campylobacter | 0 | 0 | 1 |
| c_Clostridia;o_Lachnospirales;f_Lachnospiraceae;g_Fusicatenibacter | 0 | 0 | 1 |
| c_Clostridia;o_Oscillospirales;f_Ruminococcaceae;g_Subdoligranulum | 0 | 0 | 1 |
| c_Clostridia;o_Peptostreptococcales-Tissierellales;f_Anaerovoracaceae;g_nodatum_group | 0 | 0 | 1 |
| c_Gammaproteobacteria;o_Pasteurellales;f_Pasteurellaceae;g_Actinobacillus | 0 | 0 | 1 |
| c_Clostridia;_;_;_ | 4 | 2 | 2 |
| c_Clostridia;o_Lachnospirales;f_Lachnospiraceae;g_uncultured | 3 | 3 | 2 |
| c_Clostridia;o_Peptostreptococcales-Tissierellales;f_Anaerovoracaceae;g_Family_XIII_UCG-001 | 3 | 3 | 2 |
| c_Coriobacteriia;o_Coriobacteriales;f_Eggerthellaceae;g_Enterorhabdus | 2 | 3 | 3 |
| c_Bacilli;o_Erysipelotrichales;f_Erysipelotrichaceae;g_Catenisphaera | 4 | 2 | 1 |
| c_Desulfovibrionia;o_Desulfovibrionales;f_Desulfovibrionaceae;g_Mailhella | 2 | 4 | 1 |
| c_Clostridia;o_Oscillospirales;f_Oscillospiraceae;g_Oscillibacter | 2 | 3 | 2 |
| c_Clostridia;o_Peptostreptococcales-Tissierellales;f_Peptostreptococcaceae;g_Intestinibacter | 1 | 5 | 0 |
| c_Clostridia;o_Christensenellales;f_Christensenellaceae;g_uncultured | 1 | 1 | 4 |
| c_Gammaproteobacteria;o_Burkholderiales;f_Sutterellaceae;g_Parasutterella | 4 | 1 | 0 |
| c_Lentisphaeria;o_Victivallales;f_vadinBE97;g_vadinBE97 | 1 | 2 | 2 |
| c_Clostridia;o_Oscillospirales;f_Oscillospiraceae;g_Oscillospira | 1 | 1 | 3 |
| c_Clostridia;o_Lachnospirales;f_Lachnospiraceae;g_Lachnospiraceae_FCS020_group | 2 | 2 | 0 |
| c_Deferribacteres;o_Deferribacterales;f_Deferribacteraceae;g_Mucispirillum | 1 | 3 | 0 |
| c_Alphaproteobacteria;o_Rhodospirillales;f_uncultured;g_uncultured | 1 | 3 | 0 |
| c_Bacteroidia;o_Bacteroidales;f_Tannerellaceae;g_Parabacteroides | 1 | 2 | 1 |
| c_Negativicutes;o_Veillonellales-Selenomonadales;f_Selenomonadaceae;g_Anaerovibrio | 1 | 1 | 2 |
| c_Coriobacteriia;o_Coriobacteriales;f_Eggerthellaceae;_ | 1 | 0 | 3 |
| c_Bacilli;o_Erysipelotrichales;f_Erysipelotrichaceae;g_Holdemanella | 1 | 2 | 0 |
| c_Coriobacteriia;o_Coriobacteriales;f_Eggerthellaceae;g_Slackia | 1 | 1 | 1 |
| c_Clostridia;o_Lachnospirales;f_Lachnospiraceae;g_Marvinbryantia | 0 | 3 | 0 |
| c_Bacteroidia;o_Bacteroidales;f_Prevotellaceae;g_Prevotellaceae_UCG-003 | 1 | 1 | 0 |
| c_Clostridia;o_Lachnospirales;f_Lachnospiraceae;g_Lachnospiraceae_ND3007_group | 0 | 2 | 0 |
| c_Clostridia;o_Oscillospirales;f_Oscillospiraceae;g_UCG-005 | 0 | 2 | 0 |
| c_Clostridia;o_Oscillospirales;f_Ruminococcaceae;_ | 0 | 2 | 0 |
| c_Bacteroidia;o_Bacteroidales;f_Bacteroidales_RF16_group;g_Bacteroidales_RF16_group | 0 | 1 | 1 |
| c_Thermoplasmata;o_Methanomassiliicoccales;f_Methanomethylophilaceae;g_Candidatus_Methanomethylophilus | 1 | 0 | 0 |
| c_Clostridia;o_Oscillospirales;f_Butyricicoccaceae;g_Butyricicoccus | 1 | 0 | 0 |
| c_Clostridia;o_Oscillospirales;f_Ruminococcaceae;g_Negativibacillus | 1 | 0 | 0 |
| c_Clostridia;o_Oscillospirales;f_Oscillospiraceae;g_NK4A214_group | 0 | 1 | 0 |
| c_Bacteroidia;o_Bacteroidales;f_Marinifilaceae;g_Butyricimonas | 0 | 0 | 1 |
| c_Bacilli;o_Erysipelotrichales;f_Erysipelotrichaceae;g_Erysipelotrichaceae_UCG-009 | 0 | 0 | 1 |
| c_Negativicutes;o_Veillonellales-Selenomonadales;f_Veillonellaceae;g_Allisonella | 0 | 0 | 1 |
| c_Coriobacteriia;o_Coriobacteriales;f_Atopobiaceae;g_Libanicoccus | 0 | 0 | 0 |
| c_Bacteroidia;o_Bacteroidales;f_uncultured;g_uncultured | 0 | 0 | 0 |
| c_Clostridia;o_Eubacteriales;f_Eubacteriaceae;g_Pseudoramibacter | 0 | 0 | 0 |
| c_Clostridia;o_Lachnospirales;f_Lachnospiraceae;g_eligens_group | 0 | 0 | 0 |
| c_Kiritimatiellae;o_WCHB1-41;f_WCHB1-41;g_WCHB1-41 | 2 | 4 | 3 |
| c_Lentisphaeria;o_Victivallales;f_Victivallaceae;g_Victivallaceae | 2 | 4 | 2 |
| c_Clostridia;o_Oscillospirales;f_Butyricicoccaceae;g_UCG-009 | 2 | 2 | 4 |
| c_Desulfuromonadia;o_Bradymonadales;f_Bradymonadales;g_Bradymonadales | 2 | 2 | 3 |
| c_Campylobacteria;o_Campylobacterales;f_Helicobacteraceae;g_Helicobacter | 2 | 2 | 2 |
| c_Vampirivibrionia;o_Gastranaerophilales;f_Gastranaerophilales;g_Gastranaerophilales | 2 | 2 | 2 |
| c_Bacilli;o_Erysipelotrichales;f_Erysipelatoclostridiaceae;g_UCG-004 | 3 | 1 | 1 |
| c_Clostridia;o_Oscillospirales;f_Ruminococcaceae;g_Incertae_Sedis | 2 | 3 | 0 |
| c_Clostridia;o_Lachnospirales;f_Lachnospiraceae;g_Anaerostipes | 0 | 5 | 0 |
| c_Clostridia;o_Oscillospirales;f_Ruminococcaceae;g_CAG-352 | 0 | 3 | 2 |
| c_Negativicutes;o_Veillonellales-Selenomonadales;f_Selenomonadaceae;_ | 1 | 1 | 0 |
| c_Negativicutes;o_Acidaminococcales;f_Acidaminococcaceae;g_Acidaminococcus | 1 | 0 | 1 |
| c_Negativicutes;o_Veillonellales-Selenomonadales;f_Veillonellaceae;g_Dialister | 0 | 2 | 0 |
| c_Clostridia;o_Oscillospirales;f_Oscillospiraceae;_ | 0 | 0 | 0 |
| c_Gammaproteobacteria;o_Aeromonadales;f_Succinivibrionaceae;g_Succinivibrio | 4 | 5 | 1 |
| c_Clostridia;o_Lachnospirales;f_Lachnospiraceae;g_Howardella | 3 | 5 | 1 |
| c_Clostridia;o_Lachnospirales;f_Lachnospiraceae;g_Lachnospiraceae_UCG-004 | 3 | 3 | 2 |
| c_Bacilli;o_Erysipelotrichales;f_Erysipelotrichaceae;g_Solobacterium | 3 | 2 | 2 |
| c_Coriobacteriia;o_Coriobacteriales;f_Atopobiaceae;g_Olsenella | 3 | 1 | 2 |
| c_Gammaproteobacteria;o_Burkholderiales;f_Oxalobacteraceae;_ | 3 | 5 | 2 |
| c_Clostridia;o_Oscillospirales;f_Oscillospiraceae;g_UCG-003 | 0 | 0 | 0 |

**Table 4**: Colonizing genera in all FMT treatment groups on day 5 post FMT

| Taxa | Oral | Rectal | In-feed |
| --- | --- | --- | --- |
| c_Desulfovibrionia;o_Desulfovibrionales;f_Desulfovibrionaceae;g_Mailhella | 4 | 3 | 2 |
| c_Bacilli;o_Erysipelotrichales;f_Erysipelotrichaceae;g_Solobacterium | 1 | 2 | 4 |
| c_Coriobacteriia;o_Coriobacteriales;f_Eggerthellaceae;g_Slackia | 3 | 1 | 2 |
| c_Negativicutes;o_Veillonellales-Selenomonadales;f_Veillonellaceae;g_Allisonella | 1 | 2 | 3 |
| c_Clostridia;o_Peptostreptococcales-Tissierellales;f_Anaerovoracaceae;_ | 1 | 1 | 3 |
| c_Bacteroidia;o_Bacteroidales;f_Rikenellaceae;g_dgA-11_gut_group | 1 | 1 | 2 |
| c_Deferribacteres;o_Deferribacterales;f_Deferribacteraceae;g_Mucispirillum | 1 | 1 | 2 |
| c_Bacilli;o_Erysipelotrichales;f_Erysipelotrichaceae;g_Erysipelotrichaceae_UCG-006 | 1 | 1 | 2 |
| c_Clostridia;o_Lachnospirales;f_Lachnospiraceae;g_Blautia | 3 | 1 | 0 |
| c_Clostridia;o_Lachnospirales;f_Lachnospiraceae;g_Lachnospiraceae_FCS020_group | 3 | 1 | 0 |
| c_Clostridia;o_Lachnospirales;f_Lachnospiraceae;g_Lachnospiraceae_UCG-008 | 1 | 2 | 1 |
| c_Clostridia;o_Lachnospirales;f_Lachnospiraceae;g_eligens_group | 2 | 1 | 1 |
| c_Bacteroidia;o_Bacteroidales;f_uncultured;g_uncultured | 0 | 1 | 3 |
| c_Methanobacteria;o_Methanobacteriales;f_Methanobacteriaceae;g_Methanosphaera | 1 | 1 | 1 |
| c_Thermoplasmata;o_Methanomassiliicoccales;f_Methanomethylophilaceae;g_uncultured | 1 | 2 | 0 |
| c_Bacteroidia;o_Bacteroidales;f_Prevotellaceae;g_Prevotellaceae_UCG-004 | 2 | 0 | 1 |
| c_Campylobacteria;o_Campylobacterales;f_Helicobacteraceae;g_Helicobacter | 1 | 1 | 1 |
| c_Clostridia;o_Lachnospirales;f_Lachnospiraceae;g_Howardella | 2 | 1 | 0 |
| c_Clostridia;o_Lachnospirales;f_Lachnospiraceae;g_Lachnospiraceae_NK4B4_group | 1 | 1 | 1 |
| c_Clostridia;o_Oscillospirales;f_Oscillospiraceae;g_Oscillospira | 2 | 0 | 1 |
| c_Negativicutes;o_Veillonellales-Selenomonadales;f_Selenomonadaceae;g_Anaerovibrio | 1 | 0 | 2 |
| c_Alphaproteobacteria;o_Caulobacterales;f_Caulobacteraceae;g_Brevundimonas | 1 | 1 | 1 |
| c_Actinobacteria;o_Corynebacteriales;f_Corynebacteriaceae;g_Corynebacterium | 0 | 1 | 2 |
| c_Synergistia;o_Synergistales;f_Synergistaceae;g_Pyramidobacter | 0 | 1 | 2 |
| c_Bacilli;o_Erysipelotrichales;f_Erysipelotrichaceae;g_Holdemanella | 1 | 1 | 0 |
| c_Clostridia;o_Lachnospirales;f_Lachnospiraceae;g_ventriosum_group | 1 | 0 | 1 |
| c_Clostridia;o_Lachnospirales;f_Lachnospiraceae;g_[Ruminococcus]_torques_group | 2 | 0 | 0 |
| c_Coriobacteriia;o_Coriobacteriales;f_Coriobacteriaceae;g_Collinsella | 0 | 1 | 1 |
| c_Bacteroidia;o_Bacteroidales;f_Tannerellaceae;g_Parabacteroides | 0 | 2 | 0 |
| c_Clostridia;o_Clostridia_UCG-014;f_Clostridia_UCG-014;g_Clostridia_UCG-014 | 0 | 2 | 0 |
| c_Negativicutes;o_Veillonellales-Selenomonadales;f_Veillonellaceae;g_Megasphaera | 0 | 1 | 1 |
| c_Bacteroidia;o_Bacteroidales;f_Prevotellaceae;g_uncultured | 1 | 0 | 0 |
| c_Bacilli;o_Erysipelotrichales;f_Erysipelotrichaceae;g_Turicibacter | 1 | 0 | 0 |
| c_Bacilli;o_Erysipelotrichales;f_Erysipelotrichaceae;g_uncultured | 1 | 0 | 0 |
| c_Clostridia;o_Oscillospirales;f_Oscillospiraceae;g_uncultured | 1 | 0 | 0 |
| c_Lentisphaeria;o_Victivallales;f_Victivallaceae;g_Victivallaceae | 1 | 0 | 0 |
| c_Bacteroidia;o_Bacteroidales;f_Marinifilaceae;g_Butyricimonas | 0 | 1 | 0 |
| c_Bacilli;o_Lactobacillales;f_Streptococcaceae;g_Streptococcus | 0 | 1 | 0 |
| c_Clostridia;o_Peptostreptococcales-Tissierellales;f_Peptostreptococcaceae;g_Romboutsia | 0 | 1 | 0 |
| c_Clostridia;o_Oscillospirales;f_Oscillospiraceae;g_UCG-003 | 0 | 0 | 1 |
| c_Clostridia;o_Peptostreptococcales-Tissierellales;f_Anaerovoracaceae;g_nodatum_group | 0 | 0 | 1 |
| c_Clostridia;o_Peptostreptococcales-Tissierellales;f_Peptostreptococcaceae;g_Terrisporobacter | 0 | 0 | 1 |
| c_Gammaproteobacteria;o_Pasteurellales;f_Pasteurellaceae;g_Actinobacillus | 0 | 0 | 1 |
| c_Clostridia;o_Lachnospirales;f_Lachnospiraceae;g_Lachnospiraceae_NK4A136_group | 5 | 4 | 5 |
| c_Clostridia;o_Oscillospirales;f_Butyricicoccaceae;g_UCG-008 | 4 | 3 | 4 |
| c_Fibrobacteria;o_Fibrobacterales;f_Fibrobacteraceae;g_Fibrobacter | 4 | 3 | 3 |
| c_Bacilli;o_Erysipelotrichales;f_Erysipelotrichaceae;g_Catenisphaera | 4 | 3 | 3 |
| c_Clostridia;o_Lachnospirales;f_Lachnospiraceae;g_Dorea | 4 | 1 | 4 |
| c_Clostridia;o_Lachnospirales;f_Lachnospiraceae;g_Lachnospiraceae_UCG-004 | 4 | 3 | 2 |
| c_Negativicutes;o_Veillonellales-Selenomonadales;f_Selenomonadaceae;_ | 4 | 2 | 3 |
| c_Clostridia;o_Lachnospirales;f_Lachnospiraceae;g_Lachnospiraceae_AC2044_group | 3 | 2 | 3 |
| c_Clostridia;o_Lachnospirales;f_Lachnospiraceae;g_Oribacterium | 4 | 2 | 2 |
| c_Clostridia;o_Oscillospirales;f_Ruminococcaceae;g_Faecalibacterium | 3 | 1 | 3 |
| c_Negativicutes;o_Veillonellales-Selenomonadales;f_Selenomonadaceae;g_uncultured | 2 | 2 | 3 |
| c_Negativicutes;o_Veillonellales-Selenomonadales;f_Selenomonadaceae;g_Mitsuokella | 2 | 2 | 2 |
| c_Vampirivibrionia;o_Gastranaerophilales;f_Gastranaerophilales;g_Gastranaerophilales | 3 | 1 | 1 |
| c_Desulfuromonadia;o_Bradymonadales;f_Bradymonadales;g_Bradymonadales | 2 | 1 | 2 |
| c_Clostridia;o_Lachnospirales;f_Lachnospiraceae;g_ruminantium_group | 2 | 2 | 1 |
| c_Thermoplasmata;o_Methanomassiliicoccales;f_Methanomethylophilaceae;g_Candidatus_Methanomethylophilus | 3 | 1 | 0 |
| c_Bacteroidia;o_Bacteroidales;f_Bacteroidales_RF16_group;g_Bacteroidales_RF16_group | 1 | 1 | 2 |
| c_Clostridia;o_Lachnospirales;f_Lachnospiraceae;g_hallii_group | 3 | 0 | 1 |
| c_Negativicutes;o_Veillonellales-Selenomonadales;f_Selenomonadaceae;g_Selenomonas | 1 | 0 | 3 |
| c_Bacteroidia;o_Bacteroidales;f_Prevotellaceae;g_Prevotellaceae_UCG-003 | 2 | 0 | 1 |
| c_Bacilli;o_RF39;f_RF39;g_RF39 | 2 | 1 | 0 |
| c_Clostridia;o_Lachnospirales;f_Lachnospiraceae;g_Roseburia | 3 | 0 | 0 |
| c_Negativicutes;o_Veillonellales-Selenomonadales;f_Veillonellaceae;g_Veillonella | 3 | 0 | 0 |
| c_Actinobacteria;o_Bifidobacteriales;f_Bifidobacteriaceae;g_Bifidobacterium | 0 | 1 | 1 |
| c_Coriobacteriia;o_Coriobacteriales;f_uncultured;g_uncultured | 1 | 0 | 1 |
| c_Clostridia;o_Oscillospirales;f_Oscillospiraceae;g_UCG-005 | 0 | 1 | 1 |
| c_Spirochaetia;o_Spirochaetales;f_Spirochaetaceae;g_Treponema | 1 | 0 | 1 |
| c_Coriobacteriia;o_Coriobacteriales;f_Eggerthellaceae;g_Paraeggerthella | 0 | 1 | 0 |
| c_Bacilli;o_Erysipelotrichales;f_Erysipelatoclostridiaceae;g_UCG-004 | 0 | 1 | 0 |
| c_Clostridia;o_Clostridia_vadinBB60_group;f_Clostridia_vadinBB60_group;g_Clostridia_vadinBB60_group | 0 | 1 | 0 |
| c_Clostridia;o_Oscillospirales;f_Ruminococcaceae;g_Ruminococcus | 0 | 1 | 0 |
| c_Clostridia;o_Oscillospirales;f_Ruminococcaceae;g_Subdoligranulum | 0 | 0 | 1 |
| c_Clostridia;o_Oscillospirales;f_UCG-010;g_UCG-010 | 0 | 1 | 0 |
| c_Coriobacteriia;o_Coriobacteriales;f_Atopobiaceae;g_Libanicoccus | 0 | 0 | 0 |
| c_Bacilli;o_Erysipelotrichales;f_Erysipelotrichaceae;g_Erysipelotrichaceae_UCG-009 | 0 | 0 | 0 |
| c_Clostridia;o_Lachnospirales;f_Lachnospiraceae;g_Anaerostipes | 0 | 0 | 0 |
| c_Clostridia;o_Lachnospirales;f_Lachnospiraceae;g_Shuttleworthia | 0 | 0 | 0 |
| c_Clostridia;o_Oscillospirales;f_Oscillospiraceae;g_NK4A214_group | 0 | 0 | 0 |
| c_Negativicutes;o_Veillonellales-Selenomonadales;f_Veillonellaceae;g_Dialister | 0 | 0 | 0 |
| c_Clostridia;o_Lachnospirales;f_Lachnospiraceae;g_Lachnospira | 4 | 5 | 5 |
| c_Clostridia;o_Lachnospirales;f_Lachnospiraceae;g_Agathobacter | 4 | 5 | 4 |
| c_Gammaproteobacteria;o_Burkholderiales;f_Oxalobacteraceae;_ | 4 | 4 | 2 |
| c_Coriobacteriia;o_Coriobacteriales;f_Eggerthellaceae;g_Enterorhabdus | 3 | 3 | 3 |
| c_Bacilli;o_Erysipelotrichales;f_Erysipelatoclostridiaceae;g_Catenibacterium | 5 | 2 | 2 |
| c_Thermoplasmata;o_Methanomassiliicoccales;f_Methanomethylophilaceae;g_Candidatus_Methanoplasma | 3 | 3 | 2 |
| c_Coriobacteriia;o_Coriobacteriales;f_Eggerthellaceae;_ | 4 | 3 | 1 |
| c_Coriobacteriia;o_Coriobacteriales;f_Atopobiaceae;g_Olsenella | 2 | 2 | 3 |
| c_Clostridia;o_Lachnospirales;f_Lachnospiraceae;g_Fusicatenibacter | 2 | 3 | 2 |
| c_Clostridia;o_Oscillospirales;f_Ruminococcaceae;g_Incertae_Sedis | 3 | 2 | 2 |
| c_Clostridia;o_Peptostreptococcales-Tissierellales;f_Anaerovoracaceae;g_Family_XIII_UCG-001 | 1 | 3 | 3 |
| c_Bacteroidia;o_Bacteroidales;f_Prevotellaceae;g_Prevotellaceae_UCG-001 | 2 | 2 | 1 |
| c_Clostridia;o_Lachnospirales;f_Lachnospiraceae;g_Coprococcus | 2 | 1 | 2 |
| c_Clostridia;o_Lachnospirales;f_Lachnospiraceae;g_Lachnospiraceae_UCG-003 | 0 | 2 | 3 |
| c_Alphaproteobacteria;o_Rhodospirillales;f_uncultured;g_uncultured | 3 | 2 | 0 |
| c_Clostridia;_;_;_ | 2 | 2 | 0 |
| c_Clostridia;o_Lachnospirales;f_Lachnospiraceae;g_Lachnospiraceae_UCG-007 | 1 | 2 | 1 |
| c_Negativicutes;o_Acidaminococcales;f_Acidaminococcaceae;g_Acidaminococcus | 1 | 1 | 1 |
| c_Clostridia;o_Oscillospirales;f_Ruminococcaceae;g_Fournierella | 2 | 0 | 0 |
| c_Gammaproteobacteria;o_Burkholderiales;f_Sutterellaceae;g_Parasutterella | 1 | 0 | 1 |
| c_Lentisphaeria;o_Victivallales;f_vadinBE97;g_vadinBE97 | 1 | 0 | 0 |
| c_Clostridia;o_Oscillospirales;f_Butyricicoccaceae;g_UCG-009 | 5 | 4 | 4 |
| c_Clostridia;o_Peptostreptococcales-Tissierellales;f_Peptostreptococcaceae;g_Intestinibacter | 4 | 4 | 5 |
| c_Gammaproteobacteria;o_Aeromonadales;f_Succinivibrionaceae;g_Succinivibrio | 5 | 4 | 4 |
| c_Clostridia;o_Lachnospirales;f_Lachnospiraceae;g_Lachnospiraceae_ND3007_group | 4 | 3 | 5 |
| c_Clostridia;o_Lachnospirales;f_Lachnospiraceae;g_Marvinbryantia | 5 | 3 | 4 |
| c_Clostridia;o_Oscillospirales;f_Ruminococcaceae;g_CAG-352 | 4 | 2 | 5 |
| c_Clostridia;o_Lachnospirales;f_Lachnospiraceae;g_[Ruminococcus]_gauvreauii_group | 4 | 3 | 2 |
| c_Clostridia;o_Oscillospirales;f_Ruminococcaceae;g_Negativibacillus | 4 | 3 | 2 |
| c_Kiritimatiellae;o_WCHB1-41;f_WCHB1-41;g_WCHB1-41 | 3 | 3 | 2 |
| c_Clostridia;o_Lachnospirales;f_Lachnospiraceae;g_uncultured | 2 | 2 | 1 |
| c_Clostridia;o_Oscillospirales;f_Ruminococcaceae;_ | 2 | 2 | 1 |
| c_Clostridia;o_Peptococcales;f_Peptococcaceae;g_uncultured | 2 | 0 | 2 |
| c_Clostridia;o_Lachnospirales;f_Lachnospiraceae;g_Lachnospiraceae_NK3A20_group | 1 | 1 | 1 |
| c_Clostridia;o_Oscillospirales;f_Oscillospiraceae;g_Oscillibacter | 1 | 1 | 1 |
| c_Clostridia;o_Oscillospirales;f_Oscillospiraceae;_ | 0 | 0 | 1 |
| c_Clostridia;o_Clostridiales;f_Clostridiaceae;g_Clostridium_sensu_stricto_6 | 4 | 3 | 4 |
| c_Coriobacteriia;o_Coriobacteriales;f_Atopobiaceae;g_uncultured | 2 | 3 | 4 |
| c_Clostridia;o_Monoglobales;f_Monoglobaceae;g_Monoglobus | 3 | 3 | 3 |
| c_Clostridia;o_Lachnospirales;f_Lachnospiraceae;g_xylanophilum_group | 3 | 2 | 3 |
| c_Clostridia;o_Christensenellales;f_Christensenellaceae;g_uncultured | 3 | 4 | 4 |

**Table 5**: Colonizing genera in all FMT treatment groups on day 7 post FMT

| Taxa | Oral | Rectal | In-feed |
| --- | --- | --- | --- |
| c_Coriobacteriia;o_Coriobacteriales;f_Eggerthellaceae;g_Slackia | 2 | 1 | 2 |
| c_Desulfovibrionia;o_Desulfovibrionales;f_Desulfovibrionaceae;g_Mailhella | 2 | 2 | 1 |
| c_Clostridia;o_Lachnospirales;f_Lachnospiraceae;g_Lachnospiraceae_UCG-008 | 2 | 1 | 2 |
| c_Clostridia;o_Lachnospirales;f_Lachnospiraceae;g_Blautia | 3 | 1 | 0 |
| c_Clostridia;o_Lachnospirales;f_Lachnospiraceae;g_Howardella | 2 | 2 | 0 |
| c_Clostridia;o_Lachnospirales;f_Lachnospiraceae;g_Lachnospiraceae_FCS020_group | 3 | 1 | 0 |
| c_Clostridia;o_Oscillospirales;f_Oscillospiraceae;g_Oscillospira | 2 | 0 | 2 |
| c_Bacteroidia;o_Bacteroidales;f_Prevotellaceae;g_Prevotellaceae_UCG-004 | 2 | 0 | 1 |
| c_Bacteroidia;o_Bacteroidales;f_Prevotellaceae;g_uncultured | 2 | 1 | 0 |
| c_Deferribacteres;o_Deferribacterales;f_Deferribacteraceae;g_Mucispirillum | 2 | 0 | 1 |
| c_Clostridia;o_Oscillospirales;f_Oscillospiraceae;g_uncultured | 1 | 1 | 1 |
| c_Clostridia;o_Peptostreptococcales-Tissierellales;f_Anaerovoracaceae;g_nodatum_group | 1 | 1 | 1 |
| c_Negativicutes;o_Veillonellales-Selenomonadales;f_Selenomonadaceae;g_Anaerovibrio | 1 | 0 | 2 |
| c_Actinobacteria;o_Corynebacteriales;f_Corynebacteriaceae;g_Corynebacterium | 0 | 1 | 2 |
| c_Coriobacteriia;o_Coriobacteriales;f_Coriobacteriaceae;g_Collinsella | 0 | 1 | 2 |
| c_Synergistia;o_Synergistales;f_Synergistaceae;g_Pyramidobacter | 0 | 1 | 2 |
| c_Bacteroidia;o_Bacteroidales;f_uncultured;g_uncultured | 0 | 0 | 3 |
| c_Bacilli;o_Erysipelotrichales;f_Erysipelotrichaceae;g_Erysipelotrichaceae_UCG-006 | 1 | 1 | 0 |
| c_Bacilli;o_Erysipelotrichales;f_Erysipelotrichaceae;g_Holdemanella | 1 | 1 | 0 |
| c_Clostridia;o_Lachnospirales;f_Lachnospiraceae;g_Lachnospiraceae_NK4B4_group | 2 | 0 | 0 |
| c_Clostridia;o_Lachnospirales;f_Lachnospiraceae;g_ventriosum_group | 1 | 0 | 1 |
| c_Clostridia;o_Lachnospirales;f_Lachnospiraceae;g_[Ruminococcus]_torques_group | 2 | 0 | 0 |
| c_Alphaproteobacteria;o_Caulobacterales;f_Caulobacteraceae;g_Brevundimonas | 1 | 1 | 0 |
| c_Bacteroidia;o_Bacteroidales;f_Tannerellaceae;g_Parabacteroides | 0 | 2 | 0 |
| c_Clostridia;o_Clostridia_UCG-014;f_Clostridia_UCG-014;g_Clostridia_UCG-014 | 0 | 2 | 0 |
| c_Negativicutes;o_Veillonellales-Selenomonadales;f_Veillonellaceae;g_Megasphaera | 0 | 1 | 1 |
| c_Bacilli;o_Erysipelotrichales;f_Erysipelotrichaceae;g_Turicibacter | 1 | 0 | 0 |
| c_Bacilli;o_Erysipelotrichales;f_Erysipelotrichaceae;g_uncultured | 1 | 0 | 0 |
| c_Clostridia;o_Oscillospirales;f_Butyricicoccaceae;g_Butyricicoccus | 1 | 0 | 0 |
| c_Bacteroidia;o_Bacteroidales;f_Marinifilaceae;g_Butyricimonas | 0 | 1 | 0 |
| c_Bacilli;o_Erysipelotrichales;f_Erysipelatoclostridiaceae;g_UCG-004 | 0 | 1 | 0 |
| c_Bacilli;o_Lactobacillales;f_Streptococcaceae;g_Streptococcus | 0 | 1 | 0 |
| c_Clostridia;o_Peptostreptococcales-Tissierellales;f_Peptostreptococcaceae;g_Terrisporobacter | 0 | 0 | 1 |
| c_Lentisphaeria;o_Victivallales;f_Victivallaceae;g_Victivallaceae | 0 | 0 | 1 |
| c_Fibrobacteria;o_Fibrobacterales;f_Fibrobacteraceae;g_Fibrobacter | 5 | 3 | 4 |
| c_Clostridia;o_Lachnospirales;f_Lachnospiraceae;g_Dorea | 4 | 3 | 5 |
| c_Bacilli;o_Erysipelotrichales;f_Erysipelotrichaceae;g_Catenisphaera | 3 | 2 | 3 |
| c_Clostridia;o_Lachnospirales;f_Lachnospiraceae;g_Lachnospiraceae_AC2044_group | 4 | 2 | 2 |
| c_Clostridia;o_Lachnospirales;f_Lachnospiraceae;g_Oribacterium | 4 | 2 | 2 |
| c_Clostridia;o_Oscillospirales;f_Ruminococcaceae;g_Faecalibacterium | 3 | 2 | 3 |
| c_Alphaproteobacteria;o_Rhodospirillales;f_uncultured;g_uncultured | 4 | 2 | 0 |
| c_Thermoplasmata;o_Methanomassiliicoccales;f_Methanomethylophilaceae;g_uncultured | 2 | 2 | 0 |
| c_Bacteroidia;o_Bacteroidales;f_Bacteroidales_RF16_group;g_Bacteroidales_RF16_group | 1 | 1 | 2 |
| c_Bacteroidia;o_Bacteroidales;f_Rikenellaceae;g_dgA-11_gut_group | 1 | 1 | 2 |
| c_Clostridia;o_Lachnospirales;f_Lachnospiraceae;g_hallii_group | 3 | 0 | 1 |
| c_Bacteroidia;o_Bacteroidales;f_Prevotellaceae;g_Prevotellaceae_UCG-003 | 2 | 0 | 1 |
| c_Clostridia;o_Lachnospirales;f_Lachnospiraceae;g_Roseburia | 3 | 0 | 0 |
| c_Negativicutes;o_Veillonellales-Selenomonadales;f_Veillonellaceae;g_Veillonella | 3 | 0 | 0 |
| c_Gammaproteobacteria;o_Burkholderiales;f_Sutterellaceae;g_Parasutterella | 2 | 0 | 1 |
| c_Campylobacteria;o_Campylobacterales;f_Helicobacteraceae;g_Helicobacter | 0 | 1 | 1 |
| c_Clostridia;o_Lachnospirales;f_Lachnospiraceae;g_Anaerostipes | 0 | 1 | 1 |
| c_Clostridia;o_Oscillospirales;f_Oscillospiraceae;g_UCG-005 | 0 | 1 | 1 |
| c_Spirochaetia;o_Spirochaetales;f_Spirochaetaceae;g_Treponema | 1 | 0 | 1 |
| c_Methanobacteria;o_Methanobacteriales;f_Methanobacteriaceae;g_Methanosphaera | 0 | 0 | 1 |
| c_Clostridia;o_Clostridia_vadinBB60_group;f_Clostridia_vadinBB60_group;g_Clostridia_vadinBB60_group | 0 | 1 | 0 |
| c_Clostridia;o_Oscillospirales;f_Ruminococcaceae;g_Ruminococcus | 0 | 1 | 0 |
| c_Clostridia;o_Oscillospirales;f_Ruminococcaceae;g_Subdoligranulum | 0 | 0 | 1 |
| c_Clostridia;o_Oscillospirales;f_UCG-010;g_UCG-010 | 0 | 1 | 0 |
| c_Clostridia;o_Oscillospirales;f_Oscillospiraceae;g_NK4A214_group | 0 | 0 | 0 |
| c_Negativicutes;o_Veillonellales-Selenomonadales;f_Veillonellaceae;g_Dialister | 0 | 0 | 0 |
| c_Clostridia;o_Oscillospirales;f_Butyricicoccaceae;g_UCG-008 | 4 | 4 | 5 |
| c_Clostridia;o_Lachnospirales;f_Lachnospiraceae;g_[Ruminococcus]_gauvreauii_group | 4 | 4 | 4 |
| c_Clostridia;o_Oscillospirales;f_Ruminococcaceae;g_CAG-352 | 3 | 3 | 5 |
| c_Bacilli;o_Erysipelotrichales;f_Erysipelatoclostridiaceae;g_Catenibacterium | 4 | 3 | 3 |
| c_Bacilli;o_Erysipelotrichales;f_Erysipelotrichaceae;g_Solobacterium | 2 | 3 | 5 |
| c_Coriobacteriia;o_Coriobacteriales;f_Eggerthellaceae;g_Enterorhabdus | 3 | 3 | 3 |
| c_Coriobacteriia;o_Coriobacteriales;f_Atopobiaceae;g_Olsenella | 2 | 2 | 3 |
| c_Clostridia;o_Oscillospirales;f_Ruminococcaceae;g_Incertae_Sedis | 3 | 2 | 2 |
| c_Clostridia;o_Peptostreptococcales-Tissierellales;f_Anaerovoracaceae;_ | 3 | 1 | 3 |
| c_Negativicutes;o_Veillonellales-Selenomonadales;f_Selenomonadaceae;g_Selenomonas | 1 | 1 | 4 |
| c_Bacteroidia;o_Bacteroidales;f_Prevotellaceae;g_Prevotellaceae_UCG-001 | 2 | 2 | 1 |
| c_Vampirivibrionia;o_Gastranaerophilales;f_Gastranaerophilales;g_Gastranaerophilales | 3 | 1 | 1 |
| c_Desulfuromonadia;o_Bradymonadales;f_Bradymonadales;g_Bradymonadales | 2 | 1 | 2 |
| c_Clostridia;o_Lachnospirales;f_Lachnospiraceae;g_Coprococcus | 2 | 1 | 2 |
| c_Clostridia;o_Lachnospirales;f_Lachnospiraceae;g_Lachnospiraceae_UCG-004 | 4 | 0 | 1 |
| c_Clostridia;o_Lachnospirales;f_Lachnospiraceae;g_eligens_group | 3 | 2 | 0 |
| c_Clostridia;o_Lachnospirales;f_Lachnospiraceae;g_Lachnospiraceae_UCG-007 | 1 | 2 | 1 |
| c_Clostridia;o_Lachnospirales;f_Lachnospiraceae;g_ruminantium_group | 2 | 2 | 0 |
| c_Thermoplasmata;o_Methanomassiliicoccales;f_Methanomethylophilaceae;g_Candidatus_Methanomethylophilus | 3 | 0 | 0 |
| c_Actinobacteria;o_Bifidobacteriales;f_Bifidobacteriaceae;g_Bifidobacterium | 1 | 0 | 2 |
| c_Clostridia;o_Oscillospirales;f_Ruminococcaceae;g_Fournierella | 2 | 0 | 0 |
| c_Clostridia;o_Oscillospirales;f_Oscillospiraceae;g_UCG-003 | 0 | 0 | 1 |
| c_Lentisphaeria;o_Victivallales;f_vadinBE97;g_vadinBE97 | 1 | 0 | 0 |
| c_Clostridia;o_Lachnospirales;f_Lachnospiraceae;g_Lachnospiraceae_NK4A136_group | 5 | 4 | 5 |
| c_Clostridia;o_Lachnospirales;f_Lachnospiraceae;g_Fusicatenibacter | 5 | 4 | 4 |
| c_Clostridia;o_Lachnospirales;f_Lachnospiraceae;g_Lachnospiraceae_ND3007_group | 4 | 4 | 5 |
| c_Gammaproteobacteria;o_Aeromonadales;f_Succinivibrionaceae;g_Succinivibrio | 5 | 4 | 4 |
| c_Negativicutes;o_Veillonellales-Selenomonadales;f_Selenomonadaceae;_ | 4 | 2 | 4 |
| c_Negativicutes;o_Veillonellales-Selenomonadales;f_Selenomonadaceae;g_uncultured | 2 | 4 | 4 |
| c_Gammaproteobacteria;o_Burkholderiales;f_Oxalobacteraceae;_ | 5 | 4 | 1 |
| c_Thermoplasmata;o_Methanomassiliicoccales;f_Methanomethylophilaceae;g_Candidatus_Methanoplasma | 3 | 3 | 2 |
| c_Negativicutes;o_Veillonellales-Selenomonadales;f_Selenomonadaceae;g_Mitsuokella | 3 | 3 | 2 |
| c_Coriobacteriia;o_Coriobacteriales;f_Atopobiaceae;g_uncultured | 2 | 3 | 2 |
| c_Coriobacteriia;o_Coriobacteriales;f_Eggerthellaceae;_ | 4 | 2 | 1 |
| c_Clostridia;o_Lachnospirales;f_Lachnospiraceae;g_Lachnospiraceae_NK3A20_group | 3 | 2 | 2 |
| c_Clostridia;o_Lachnospirales;f_Lachnospiraceae;g_Lachnospiraceae_UCG-003 | 2 | 3 | 2 |
| c_Clostridia;_;_;_ | 2 | 1 | 2 |
| c_Clostridia;o_Lachnospirales;f_Lachnospiraceae;g_uncultured | 2 | 2 | 1 |
| c_Clostridia;o_Oscillospirales;f_Ruminococcaceae;_ | 2 | 2 | 1 |
| c_Clostridia;o_Peptostreptococcales-Tissierellales;f_Anaerovoracaceae;g_Family_XIII_UCG-001 | 1 | 2 | 2 |
| c_Negativicutes;o_Veillonellales-Selenomonadales;f_Veillonellaceae;g_Allisonella | 1 | 2 | 2 |
| c_Bacilli;o_RF39;f_RF39;g_RF39 | 3 | 1 | 0 |
| c_Clostridia;o_Peptococcales;f_Peptococcaceae;g_uncultured | 1 | 1 | 2 |
| c_Coriobacteriia;o_Coriobacteriales;f_uncultured;g_uncultured | 1 | 1 | 1 |
| c_Clostridia;o_Oscillospirales;f_Oscillospiraceae;g_Oscillibacter | 1 | 1 | 1 |
| c_Negativicutes;o_Acidaminococcales;f_Acidaminococcaceae;g_Acidaminococcus | 1 | 1 | 1 |
| c_Clostridia;o_Oscillospirales;f_Oscillospiraceae;_ | 0 | 0 | 1 |
| c_Coriobacteriia;o_Coriobacteriales;f_Atopobiaceae;g_Libanicoccus | 0 | 0 | 0 |
| c_Clostridia;o_Lachnospirales;f_Lachnospiraceae;g_Agathobacter | 5 | 5 | 5 |
| c_Clostridia;o_Lachnospirales;f_Lachnospiraceae;g_Marvinbryantia | 5 | 4 | 5 |
| c_Clostridia;o_Oscillospirales;f_Butyricicoccaceae;g_UCG-009 | 5 | 4 | 4 |
| c_Clostridia;o_Oscillospirales;f_Ruminococcaceae;g_Negativibacillus | 5 | 5 | 3 |
| c_Clostridia;o_Christensenellales;f_Christensenellaceae;g_uncultured | 4 | 4 | 2 |
| c_Clostridia;o_Clostridiales;f_Clostridiaceae;g_Clostridium_sensu_stricto_6 | 3 | 3 | 3 |
| c_Clostridia;o_Monoglobales;f_Monoglobaceae;g_Monoglobus | 3 | 3 | 3 |
| c_Clostridia;o_Lachnospirales;f_Lachnospiraceae;g_Lachnospira | 4 | 3 | 1 |
| c_Kiritimatiellae;o_WCHB1-41;f_WCHB1-41;g_WCHB1-41 | 3 | 2 | 2 |
| c_Clostridia;o_Peptostreptococcales-Tissierellales;f_Peptostreptococcaceae;g_Intestinibacter | 3 | 5 | 5 |
| c_Clostridia;o_Lachnospirales;f_Lachnospiraceae;g_xylanophilum_group | 3 | 4 | 2 |

**Table 6**: Rejected genera in all FMT treatment groups on day 2 post FMT

| Taxa | Oral | Rectal | In-feed | |
| --- | --- | --- | --- | --- |
| c__Methanobacteria;o__Methanobacteriales;f__Methanobacteriaceae;g__Methanosphaera | 2 | 3 | 5 |  |
| c__Thermoplasmata;o__Methanomassiliicoccales;f__Methanomethylophilaceae;g__Candidatus_Methanomethylophilus | 2 | 2 | 1 |  |
| c__Thermoplasmata;o__Methanomassiliicoccales;f__Methanomethylophilaceae;g__Candidatus_Methanoplasma | 2 | 3 | 2 |  |
| c__Thermoplasmata;o__Methanomassiliicoccales;f__Methanomethylophilaceae;g__uncultured | 2 | 2 | 0 |  |
| c__Actinobacteria;o__Bifidobacteriales;f__Bifidobacteriaceae;g__Bifidobacterium | 3 | 3 | 4 |  |
| c__Coriobacteriia;o__Coriobacteriales;f__Atopobiaceae;g__Libanicoccus | 9 | 10 | 10 |  |
| c__Coriobacteriia;o__Coriobacteriales;f__Atopobiaceae;g__Olsenella | 3 | 5 | 2 |  |
| c__Coriobacteriia;o__Coriobacteriales;f__Atopobiaceae;g__uncultured | 3 | 5 | 7 |  |
| c__Coriobacteriia;o__Coriobacteriales;f__Eggerthellaceae;__ | 6 | 8 | 6 |  |
| c__Coriobacteriia;o__Coriobacteriales;f__Eggerthellaceae;g__Enterorhabdus | 3 | 4 | 4 |  |
| c__Coriobacteriia;o__Coriobacteriales;f__Eggerthellaceae;g__Paraeggerthella | 4 | 8 | 4 |  |
| c__Coriobacteriia;o__Coriobacteriales;f__Eggerthellaceae;g__Senegalimassilia | 10 | 9 | 9 |  |
| c__Coriobacteriia;o__Coriobacteriales;f__Eggerthellaceae;g__Slackia | 5 | 2 | 7 |  |
| c__Coriobacteriia;o__Coriobacteriales;f__uncultured;g__uncultured | 8 | 8 | 9 |  |
| c__Bacteroidia;o__Bacteroidales;f__Bacteroidales_RF16_group;g__Bacteroidales_RF16_group | 6 | 7 | 9 |  |
| c__Bacteroidia;o__Bacteroidales;f__Prevotellaceae;g__Prevotellaceae_UCG-001 | 1 | 2 | 1 |  |
| c__Bacteroidia;o__Bacteroidales;f__Prevotellaceae;g__Prevotellaceae_UCG-003 | 1 | 0 | 1 |  |
| c__Bacteroidia;o__Bacteroidales;f__Prevotellaceae;g__Prevotellaceae_UCG-004 | 1 | 1 | 1 |  |
| c__Bacteroidia;o__Bacteroidales;f__Rikenellaceae;g__dgA-11_gut_group | 0 | 4 | 2 |  |
| c__Bacteroidia;o__Bacteroidales;f__uncultured;g__uncultured | 7 | 8 | 8 |  |
| c__Vampirivibrionia;o__Gastranaerophilales;f__Gastranaerophilales;g__Gastranaerophilales | 1 | 2 | 1 |  |
| c__Deferribacteres;o__Deferribacterales;f__Deferribacteraceae;g__Mucispirillum | 7 | 5 | 7 |  |
| c__Desulfovibrionia;o__Desulfovibrionales;f__Desulfovibrionaceae;g__Mailhella | 3 | 0 | 1 |  |
| c__Desulfuromonadia;o__Bradymonadales;f__Bradymonadales;g__Bradymonadales | 2 | 1 | 3 |  |
| c__Fibrobacteria;o__Fibrobacterales;f__Fibrobacteraceae;g__Fibrobacter | 5 | 6 | 10 |  |
| c__Bacilli;o__Erysipelotrichales;f__Erysipelatoclostridiaceae;g__Catenibacterium | 5 | 2 | 5 |  |
| c__Bacilli;o__Erysipelotrichales;f__Erysipelatoclostridiaceae;g__UCG-004 | 0 | 2 | 1 |  |
| c__Bacilli;o__Erysipelotrichales;f__Erysipelotrichaceae;g__Catenisphaera | 4 | 6 | 7 |  |
| c__Bacilli;o__Erysipelotrichales;f__Erysipelotrichaceae;g__Erysipelotrichaceae_UCG-006 | 7 | 3 | 6 |  |
| c__Bacilli;o__Erysipelotrichales;f__Erysipelotrichaceae;g__Erysipelotrichaceae_UCG-009 | 10 | 10 | 9 |  |
| c__Bacilli;o__Erysipelotrichales;f__Erysipelotrichaceae;g__Solobacterium | 4 | 6 | 8 |  |
| c__Bacilli;o__RF39;f__RF39;g__RF39 | 2 | 3 | 1 |  |
| c__Clostridia;__;__;__ | 1 | 5 | 3 |  |
| c__Clostridia;o__Christensenellales;f__Christensenellaceae;g__uncultured | 5 | 5 | 3 |  |
| c__Clostridia;o__Clostridia_vadinBB60_group;f__Clostridia_vadinBB60_group;g__Clostridia_vadinBB60_group | 0 | 1 | 0 |  |
| c__Clostridia;o__Clostridiales;f__Clostridiaceae;g__Clostridium_sensu_stricto_6 | 9 | 9 | 10 |  |
| c__Clostridia;o__Eubacteriales;f__Eubacteriaceae;g__Pseudoramibacter | 10 | 10 | 8 |  |
| c__Clostridia;o__Lachnospirales;f__Lachnospiraceae;g__Agathobacter | 10 | 10 | 10 |  |
| c__Clostridia;o__Lachnospirales;f__Lachnospiraceae;g__Anaerostipes | 9 | 5 | 8 |  |
| c__Clostridia;o__Lachnospirales;f__Lachnospiraceae;g__Butyrivibrio | 10 | 10 | 10 |  |
| c__Clostridia;o__Lachnospirales;f__Lachnospiraceae;g__Coprococcus | 1 | 2 | 4 |  |
| c__Clostridia;o__Lachnospirales;f__Lachnospiraceae;g__Dorea | 8 | 9 | 10 |  |
| c__Clostridia;o__Lachnospirales;f__Lachnospiraceae;g__Fusicatenibacter | 10 | 9 | 9 |  |
| c__Clostridia;o__Lachnospirales;f__Lachnospiraceae;g__Lachnospira | 10 | 10 | 10 |  |
| c__Clostridia;o__Lachnospirales;f__Lachnospiraceae;g__Lachnospiraceae_AC2044_group | 8 | 9 | 8 |  |
| c__Clostridia;o__Lachnospirales;f__Lachnospiraceae;g__Lachnospiraceae_ND3007_group | 6 | 7 | 10 |  |
| c__Clostridia;o__Lachnospirales;f__Lachnospiraceae;g__Lachnospiraceae_NK3A20_group | 9 | 8 | 10 |  |
| c__Clostridia;o__Lachnospirales;f__Lachnospiraceae;g__Lachnospiraceae_NK4A136_group | 8 | 8 | 9 |  |
| c__Clostridia;o__Lachnospirales;f__Lachnospiraceae;g__Lachnospiraceae_NK4B4_group | 10 | 10 | 10 |  |
| c__Clostridia;o__Lachnospirales;f__Lachnospiraceae;g__Lachnospiraceae_UCG-003 | 10 | 10 | 10 |  |
| c__Clostridia;o__Lachnospirales;f__Lachnospiraceae;g__Lachnospiraceae_UCG-004 | 3 | 2 | 4 |  |
| c__Clostridia;o__Lachnospirales;f__Lachnospiraceae;g__Lachnospiraceae_UCG-007 | 9 | 8 | 10 |  |
| c__Clostridia;o__Lachnospirales;f__Lachnospiraceae;g__Lachnospiraceae_UCG-008 | 10 | 8 | 9 |  |
| c__Clostridia;o__Lachnospirales;f__Lachnospiraceae;g__Marvinbryantia | 7 | 5 | 10 |  |
| c__Clostridia;o__Lachnospirales;f__Lachnospiraceae;g__Oribacterium | 1 | 4 | 2 |  |
| c__Clostridia;o__Lachnospirales;f__Lachnospiraceae;g__Roseburia | 0 | 0 | 1 |  |
| c__Clostridia;o__Lachnospirales;f__Lachnospiraceae;g__Shuttleworthia | 9 | 10 | 10 |  |
| c__Clostridia;o__Lachnospirales;f__Lachnospiraceae;g__Syntrophococcus | 10 | 10 | 10 |  |
| c__Clostridia;o__Lachnospirales;f__Lachnospiraceae;g__[Eubacterium]_eligens_group | 10 | 10 | 10 |  |
| c__Clostridia;o__Lachnospirales;f__Lachnospiraceae;g__[Eubacterium]_hallii_group | 2 | 1 | 4 |  |
| c__Clostridia;o__Lachnospirales;f__Lachnospiraceae;g__[Eubacterium]_ruminantium_group | 10 | 8 | 10 |  |
| c__Clostridia;o__Lachnospirales;f__Lachnospiraceae;g__[Eubacterium]_ventriosum_group | 10 | 9 | 10 |  |
| c__Clostridia;o__Lachnospirales;f__Lachnospiraceae;g__[Eubacterium]_xylanophilum_group | 9 | 8 | 10 |  |
| c__Clostridia;o__Lachnospirales;f__Lachnospiraceae;g__[Ruminococcus]_gauvreauii_group | 8 | 5 | 8 |  |
| c__Clostridia;o__Lachnospirales;f__Lachnospiraceae;g__uncultured | 2 | 3 | 2 |  |
| c__Clostridia;o__Monoglobales;f__Monoglobaceae;g__Monoglobus | 2 | 4 | 4 |  |
| c__Clostridia;o__Oscillospirales;f__Butyricicoccaceae;g__UCG-008 | 7 | 6 | 10 |  |
| c__Clostridia;o__Oscillospirales;f__Butyricicoccaceae;g__UCG-009 | 6 | 7 | 5 |  |
| c__Clostridia;o__Oscillospirales;f__Oscillospiraceae;__ | 0 | 0 | 1 |  |
| c__Clostridia;o__Oscillospirales;f__Oscillospiraceae;g__Oscillibacter | 0 | 1 | 0 |  |
| c__Clostridia;o__Oscillospirales;f__Oscillospiraceae;g__Oscillospira | 1 | 2 | 0 |  |
| c__Clostridia;o__Oscillospirales;f__Oscillospiraceae;g__UCG-003 | 1 | 2 | 2 |  |
| c__Clostridia;o__Oscillospirales;f__Ruminococcaceae;__ | 5 | 3 | 2 |  |
| c__Clostridia;o__Oscillospirales;f__Ruminococcaceae;g__CAG-352 | 5 | 6 | 8 |  |
| c__Clostridia;o__Oscillospirales;f__Ruminococcaceae;g__Faecalibacterium | 2 | 4 | 5 |  |
| c__Clostridia;o__Oscillospirales;f__Ruminococcaceae;g__Fournierella | 0 | 0 | 2 |  |
| c__Clostridia;o__Oscillospirales;f__Ruminococcaceae;g__Incertae_Sedis | 3 | 2 | 3 |  |
| c__Clostridia;o__Oscillospirales;f__Ruminococcaceae;g__Negativibacillus | 9 | 10 | 9 |  |
| c__Clostridia;o__Oscillospirales;f__Ruminococcaceae;g__Paludicola | 10 | 10 | 10 |  |
| c__Clostridia;o__Oscillospirales;f__Ruminococcaceae;g__Ruminococcus | 0 | 1 | 0 |  |
| c__Clostridia;o__Oscillospirales;f__Ruminococcaceae;g__Subdoligranulum | 0 | 0 | 1 |  |
| c__Clostridia;o__Oscillospirales;f__UCG-010;g__UCG-010 | 0 | 1 | 0 |  |
| c__Clostridia;o__Peptococcales;f__Peptococcaceae;g__uncultured | 3 | 4 | 1 |  |
| c__Clostridia;o__Peptostreptococcales-Tissierellales;f__Anaerovoracaceae;__ | 7 | 9 | 10 |  |
| c__Clostridia;o__Peptostreptococcales-Tissierellales;f__Anaerovoracaceae;g__Family_XIII_UCG-001 | 0 | 3 | 5 |  |
| c__Clostridia;o__Peptostreptococcales-Tissierellales;f__Peptostreptococcaceae;g__Intestinibacter | 7 | 4 | 10 |  |
| c__Clostridia;o__Peptostreptococcales-Tissierellales;f__Peptostreptococcaceae;g__Romboutsia | 0 | 0 | 0 |  |
| c__Negativicutes;o__Acidaminococcales;f__Acidaminococcaceae;g__Acidaminococcus | 6 | 4 | 6 |  |
| c__Negativicutes;o__Veillonellales-Selenomonadales;f__Selenomonadaceae;__ | 6 | 6 | 9 |  |
| c__Negativicutes;o__Veillonellales-Selenomonadales;f__Selenomonadaceae;g__Mitsuokella | 10 | 9 | 9 |  |
| c__Negativicutes;o__Veillonellales-Selenomonadales;f__Selenomonadaceae;g__Selenomonas | 9 | 9 | 9 |  |
| c__Negativicutes;o__Veillonellales-Selenomonadales;f__Selenomonadaceae;g__uncultured | 7 | 6 | 10 |  |
| c__Negativicutes;o__Veillonellales-Selenomonadales;f__Veillonellaceae;g__Allisonella | 5 | 8 | 7 |  |
| c__Negativicutes;o__Veillonellales-Selenomonadales;f__Veillonellaceae;g__Dialister | 7 | 5 | 10 |  |
| c__Negativicutes;o__Veillonellales-Selenomonadales;f__Veillonellaceae;g__Veillonella | 2 | 2 | 1 |  |
| c__Alphaproteobacteria;o__Rhodospirillales;f__uncultured;g__uncultured | 3 | 4 | 7 |  |
| c__Gammaproteobacteria;o__Aeromonadales;f__Succinivibrionaceae;__ | 10 | 10 | 10 |  |
| c__Gammaproteobacteria;o__Aeromonadales;f__Succinivibrionaceae;g__Succinivibrio | 3 | 4 | 8 |  |
| c__Gammaproteobacteria;o__Aeromonadales;f__Succinivibrionaceae;g__Succinivibrionaceae_UCG-001 | 10 | 10 | 10 |  |
| c__Gammaproteobacteria;o__Burkholderiales;f__Oxalobacteraceae;__ | 4 | 1 | 3 |  |
| c__Gammaproteobacteria;o__Burkholderiales;f__Sutterellaceae;g__Parasutterella | 2 | 7 | 7 |  |
| c__Spirochaetia;o__Spirochaetales;f__Spirochaetaceae;g__Treponema | 0 | 0 | 2 |  |
| c__Kiritimatiellae;o__WCHB1-41;f__WCHB1-41;g__WCHB1-41 | 1 | 4 | 4 |  |
| c__Lentisphaeria;o__Victivallales;f__Victivallaceae;g__Victivallaceae | 2 | 0 | 1 |  |
| c__Lentisphaeria;o__Victivallales;f__vadinBE97;g__vadinBE97 | 2 | 1 | 0 |  |
| c__Bacteroidia;o__Bacteroidales;f__Prevotellaceae;g__uncultured | 2 | 1 | 1 |  |
| c__Bacilli;o__Erysipelotrichales;f__Erysipelotrichaceae;g__uncultured | 1 | 0 | 0 |  |
| c__Clostridia;o__Lachnospirales;f__Lachnospiraceae;g__Howardella | 1 | 1 | 1 |  |
| c__Clostridia;o__Lachnospirales;f__Lachnospiraceae;g__Lachnospiraceae_FCS020_group | 2 | 0 | 3 |  |
| c__Clostridia;o__Peptostreptococcales-Tissierellales;f__Anaerovoracaceae;g__[Eubacterium]_nodatum_group | 1 | 1 | 1 |  |
| c__Alphaproteobacteria;o__Caulobacterales;f__Caulobacteraceae;g__Brevundimonas | 2 | 3 | 1 |  |
| c__Actinobacteria;o__Corynebacteriales;f__Corynebacteriaceae;g__Corynebacterium | 0 | 1 | 1 |  |
| c__Bacteroidia;o__Bacteroidales;f__Marinifilaceae;g__Butyricimonas | 0 | 1 | 0 |  |
| c__Clostridia;o__Clostridia_UCG-014;f__Clostridia_UCG-014;g__Clostridia_UCG-014 | 0 | 3 | 1 |  |
| c__Clostridia;o__Oscillospirales;f__Oscillospiraceae;g__UCG-005 | 0 | 1 | 2 |  |
| c__Clostridia;o__Oscillospirales;f__Oscillospiraceae;g__uncultured | 0 | 2 | 0 |  |
| c__Negativicutes;o__Veillonellales-Selenomonadales;f__Selenomonadaceae;g__Anaerovibrio | 0 | 1 | 1 |  |
| c__Negativicutes;o__Veillonellales-Selenomonadales;f__Veillonellaceae;g__Megasphaera | 0 | 1 | 0 |  |
| c__Clostridia;o__Peptococcales;f__Peptococcaceae;g__Peptococcus | 0 | 0 | 2 |  |

| **Table 7**: Rejected genera in all FMT treatment groups on day 5 post FMT |  |  |  |
| --- | --- | --- | --- |
| Taxa | Oral | Rectal | In-feed |
| c__Methanobacteria;o__Methanobacteriales;f__Methanobacteriaceae;g__Methanosphaera | 0 | 3 | 1 |
| c__Thermoplasmata;o__Methanomassiliicoccales;f__Methanomethylophilaceae;g__Candidatus_Methanomethylophilus | 0 | 0 | 0 |
| c__Thermoplasmata;o__Methanomassiliicoccales;f__Methanomethylophilaceae;g__Candidatus_Methanoplasma | 0 | 0 | 0 |
| c__Thermoplasmata;o__Methanomassiliicoccales;f__Methanomethylophilaceae;g__uncultured | 1 | 0 | 0 |
| c__Actinobacteria;o__Bifidobacteriales;f__Bifidobacteriaceae;g__Bifidobacterium | 1 | 1 | 1 |
| c__Coriobacteriia;o__Coriobacteriales;f__Atopobiaceae;g__Libanicoccus | 5 | 5 | 5 |
| c__Coriobacteriia;o__Coriobacteriales;f__Eggerthellaceae;__ | 1 | 1 | 3 |
| c__Coriobacteriia;o__Coriobacteriales;f__Eggerthellaceae;g__Paraeggerthella | 0 | 4 | 2 |
| c__Coriobacteriia;o__Coriobacteriales;f__Eggerthellaceae;g__Senegalimassilia | 5 | 5 | 5 |
| c__Coriobacteriia;o__Coriobacteriales;f__uncultured;g__uncultured | 3 | 4 | 4 |
| c__Bacteroidia;o__Bacteroidales;f__Bacteroidales_RF16_group;g__Bacteroidales_RF16_group | 1 | 2 | 3 |
| c__Bacteroidia;o__Bacteroidales;f__Rikenellaceae;g__dgA-11_gut_group | 0 | 0 | 0 |
| c__Bacteroidia;o__Bacteroidales;f__uncultured;g__uncultured | 4 | 4 | 1 |
| c__Campylobacteria;o__Campylobacterales;f__Helicobacteraceae;g__Helicobacter | 0 | 0 | 0 |
| c__Vampirivibrionia;o__Gastranaerophilales;f__Gastranaerophilales;g__Gastranaerophilales | 0 | 0 | 0 |
| c__Deferribacteres;o__Deferribacterales;f__Deferribacteraceae;g__Mucispirillum | 3 | 2 | 2 |
| c__Desulfuromonadia;o__Bradymonadales;f__Bradymonadales;g__Bradymonadales | 0 | 0 | 1 |
| c__Fibrobacteria;o__Fibrobacterales;f__Fibrobacteraceae;g__Fibrobacter | 1 | 0 | 2 |
| c__Bacilli;o__Erysipelotrichales;f__Erysipelatoclostridiaceae;g__UCG-004 | 1 | 0 | 0 |
| c__Bacilli;o__Erysipelotrichales;f__Erysipelotrichaceae;g__Catenisphaera | 0 | 0 | 2 |
| c__Bacilli;o__Erysipelotrichales;f__Erysipelotrichaceae;g__Erysipelotrichaceae_UCG-006 | 4 | 1 | 1 |
| c__Bacilli;o__Erysipelotrichales;f__Erysipelotrichaceae;g__Erysipelotrichaceae_UCG-009 | 5 | 5 | 5 |
| c__Bacilli;o__Erysipelotrichales;f__Erysipelotrichaceae;g__Solobacterium | 2 | 1 | 1 |
| c__Bacilli;o__RF39;f__RF39;g__RF39 | 1 | 0 | 0 |
| c__Clostridia;__;__;__ | 1 | 1 | 2 |
| c__Clostridia;o__Clostridiales;f__Clostridiaceae;g__Clostridium_sensu_stricto_6 | 1 | 2 | 1 |
| c__Clostridia;o__Eubacteriales;f__Eubacteriaceae;g__Pseudoramibacter | 5 | 5 | 4 |
| c__Clostridia;o__Lachnospirales;f__Lachnospiraceae;g__Agathobacter | 1 | 0 | 1 |
| c__Clostridia;o__Lachnospirales;f__Lachnospiraceae;g__Anaerostipes | 5 | 5 | 4 |
| c__Clostridia;o__Lachnospirales;f__Lachnospiraceae;g__Butyrivibrio | 5 | 5 | 5 |
| c__Clostridia;o__Lachnospirales;f__Lachnospiraceae;g__Dorea | 0 | 3 | 1 |
| c__Clostridia;o__Lachnospirales;f__Lachnospiraceae;g__Fusicatenibacter | 3 | 1 | 3 |
| c__Clostridia;o__Lachnospirales;f__Lachnospiraceae;g__Howardella | 1 | 2 | 2 |
| c__Clostridia;o__Lachnospirales;f__Lachnospiraceae;g__Lachnospira | 1 | 0 | 0 |
| c__Clostridia;o__Lachnospirales;f__Lachnospiraceae;g__Lachnospiraceae_AC2044_group | 2 | 2 | 2 |
| c__Clostridia;o__Lachnospirales;f__Lachnospiraceae;g__Lachnospiraceae_NK3A20_group | 4 | 4 | 4 |
| c__Clostridia;o__Lachnospirales;f__Lachnospiraceae;g__Lachnospiraceae_NK4A136_group | 0 | 0 | 0 |
| c__Clostridia;o__Lachnospirales;f__Lachnospiraceae;g__Lachnospiraceae_NK4B4_group | 4 | 4 | 4 |
| c__Clostridia;o__Lachnospirales;f__Lachnospiraceae;g__Lachnospiraceae_UCG-003 | 5 | 3 | 2 |
| c__Clostridia;o__Lachnospirales;f__Lachnospiraceae;g__Lachnospiraceae_UCG-004 | 1 | 0 | 0 |
| c__Clostridia;o__Lachnospirales;f__Lachnospiraceae;g__Lachnospiraceae_UCG-007 | 3 | 2 | 4 |
| c__Clostridia;o__Lachnospirales;f__Lachnospiraceae;g__Lachnospiraceae_UCG-008 | 4 | 3 | 4 |
| c__Clostridia;o__Lachnospirales;f__Lachnospiraceae;g__Marvinbryantia | 0 | 1 | 1 |
| c__Clostridia;o__Lachnospirales;f__Lachnospiraceae;g__Shuttleworthia | 5 | 5 | 5 |
| c__Clostridia;o__Lachnospirales;f__Lachnospiraceae;g__Syntrophococcus | 5 | 5 | 5 |
| c__Clostridia;o__Lachnospirales;f__Lachnospiraceae;g__[Eubacterium]_eligens_group | 3 | 4 | 4 |
| c__Clostridia;o__Lachnospirales;f__Lachnospiraceae;g__[Eubacterium]_ruminantium_group | 3 | 3 | 4 |
| c__Clostridia;o__Lachnospirales;f__Lachnospiraceae;g__[Eubacterium]_ventriosum_group | 4 | 5 | 4 |
| c__Clostridia;o__Lachnospirales;f__Lachnospiraceae;g__[Eubacterium]_xylanophilum_group | 2 | 3 | 2 |
| c__Clostridia;o__Lachnospirales;f__Lachnospiraceae;g__[Ruminococcus]_gauvreauii_group | 0 | 1 | 2 |
| c__Clostridia;o__Oscillospirales;f__Butyricicoccaceae;g__UCG-008 | 0 | 1 | 1 |
| c__Clostridia;o__Oscillospirales;f__Butyricicoccaceae;g__UCG-009 | 0 | 0 | 0 |
| c__Clostridia;o__Oscillospirales;f__Oscillospiraceae;g__UCG-003 | 0 | 0 | 0 |
| c__Clostridia;o__Oscillospirales;f__Ruminococcaceae;g__CAG-352 | 0 | 2 | 0 |
| c__Clostridia;o__Oscillospirales;f__Ruminococcaceae;g__Negativibacillus | 1 | 2 | 2 |
| c__Clostridia;o__Oscillospirales;f__Ruminococcaceae;g__Paludicola | 5 | 5 | 5 |
| c__Clostridia;o__Peptostreptococcales-Tissierellales;f__Anaerovoracaceae;__ | 4 | 4 | 2 |
| c__Clostridia;o__Peptostreptococcales-Tissierellales;f__Anaerovoracaceae;g__Family_XIII_UCG-001 | 0 | 0 | 0 |
| c__Clostridia;o__Peptostreptococcales-Tissierellales;f__Peptostreptococcaceae;g__Intestinibacter | 0 | 1 | 0 |
| c__Clostridia;o__Peptostreptococcales-Tissierellales;f__Peptostreptococcaceae;g__Romboutsia | 0 | 0 | 0 |
| c__Negativicutes;o__Acidaminococcales;f__Acidaminococcaceae;g__Acidaminococcus | 2 | 1 | 2 |
| c__Negativicutes;o__Veillonellales-Selenomonadales;f__Selenomonadaceae;__ | 1 | 1 | 1 |
| c__Negativicutes;o__Veillonellales-Selenomonadales;f__Selenomonadaceae;g__Mitsuokella | 3 | 3 | 3 |
| c__Negativicutes;o__Veillonellales-Selenomonadales;f__Selenomonadaceae;g__Selenomonas | 4 | 4 | 2 |
| c__Negativicutes;o__Veillonellales-Selenomonadales;f__Selenomonadaceae;g__uncultured | 2 | 2 | 2 |
| c__Negativicutes;o__Veillonellales-Selenomonadales;f__Veillonellaceae;g__Allisonella | 1 | 2 | 1 |
| c__Negativicutes;o__Veillonellales-Selenomonadales;f__Veillonellaceae;g__Dialister | 2 | 3 | 5 |
| c__Alphaproteobacteria;o__Rhodospirillales;f__uncultured;g__uncultured | 1 | 1 | 3 |
| c__Gammaproteobacteria;o__Aeromonadales;f__Succinivibrionaceae;__ | 5 | 5 | 5 |
| c__Gammaproteobacteria;o__Aeromonadales;f__Succinivibrionaceae;g__Succinivibrionaceae_UCG-001 | 5 | 5 | 5 |
| c__Gammaproteobacteria;o__Burkholderiales;f__Oxalobacteraceae;__ | 1 | 0 | 0 |
| c__Gammaproteobacteria;o__Burkholderiales;f__Sutterellaceae;g__Parasutterella | 2 | 5 | 2 |
| c__Kiritimatiellae;o__WCHB1-41;f__WCHB1-41;g__WCHB1-41 | 0 | 0 | 1 |
| c__Lentisphaeria;o__Victivallales;f__Victivallaceae;g__Victivallaceae | 1 | 2 | 2 |
| c__Lentisphaeria;o__Victivallales;f__vadinBE97;g__vadinBE97 | 1 | 1 | 0 |
| c__Bacteroidia;o__Bacteroidales;f__Prevotellaceae;g__uncultured | 1 | 1 | 0 |
| c__Clostridia;o__Christensenellales;f__Christensenellaceae;g__uncultured | 1 | 0 | 0 |
| c__Clostridia;o__Oscillospirales;f__Butyricicoccaceae;g__Butyricicoccus | 1 | 0 | 0 |
| c__Clostridia;o__Peptostreptococcales-Tissierellales;f__Anaerovoracaceae;g__[Eubacterium]_nodatum_group | 1 | 1 | 0 |
| c__Actinobacteria;o__Corynebacteriales;f__Corynebacteriaceae;g__Corynebacterium | 0 | 1 | 0 |
| c__Bacilli;o__Erysipelotrichales;f__Erysipelatoclostridiaceae;g__Catenibacterium | 0 | 2 | 2 |
| c__Clostridia;o__Lachnospirales;f__Lachnospiraceae;g__Lachnospiraceae_ND3007_group | 0 | 1 | 0 |
| c__Clostridia;o__Oscillospirales;f__Oscillospiraceae;g__uncultured | 0 | 1 | 1 |
| c__Clostridia;o__Oscillospirales;f__Ruminococcaceae;g__Faecalibacterium | 0 | 1 | 0 |
| c__Clostridia;o__Peptococcales;f__Peptococcaceae;g__uncultured | 0 | 1 | 0 |
| c__Coriobacteriia;o__Coriobacteriales;f__Coriobacteriaceae;g__Collinsella | 0 | 0 | 1 |
| c__Coriobacteriia;o__Coriobacteriales;f__Eggerthellaceae;g__Slackia | 0 | 0 | 2 |
| c__Bacilli;o__Erysipelotrichales;f__Erysipelotrichaceae;g__Turicibacter | 0 | 0 | 1 |
| c__Clostridia;o__Oscillospirales;f__Oscillospiraceae;g__Oscillospira | 0 | 0 | 1 |
| c__Clostridia;o__Peptococcales;f__Peptococcaceae;g__Peptococcus | 0 | 0 | 1 |
| c__Gammaproteobacteria;o__Aeromonadales;f__Succinivibrionaceae;g__Succinivibrio | 0 | 0 | 1 |

**Table 8:** Rejected genera in all FMT treatment groups on day 7 post FMT

| Taxa | Oral | Rectal | In-feed |
| --- | --- | --- | --- |
| c__Methanobacteria;o__Methanobacteriales;f__Methanobacteriaceae;g__Methanosphaera | 0 | 3 | 1 |
| c__Thermoplasmata;o__Methanomassiliicoccales;f__Methanomethylophilaceae;g__Candidatus_Methanomethylophilus | 0 | 0 | 0 |
| c__Thermoplasmata;o__Methanomassiliicoccales;f__Methanomethylophilaceae;g__Candidatus_Methanoplasma | 0 | 0 | 0 |
| c__Thermoplasmata;o__Methanomassiliicoccales;f__Methanomethylophilaceae;g__uncultured | 1 | 0 | 0 |
| c__Actinobacteria;o__Bifidobacteriales;f__Bifidobacteriaceae;g__Bifidobacterium | 1 | 1 | 1 |
| c__Coriobacteriia;o__Coriobacteriales;f__Atopobiaceae;g__Libanicoccus | 5 | 5 | 5 |
| c__Coriobacteriia;o__Coriobacteriales;f__Eggerthellaceae;__ | 1 | 1 | 3 |
| c__Coriobacteriia;o__Coriobacteriales;f__Eggerthellaceae;g__Paraeggerthella | 0 | 4 | 2 |
| c__Coriobacteriia;o__Coriobacteriales;f__Eggerthellaceae;g__Senegalimassilia | 5 | 5 | 5 |
| c__Coriobacteriia;o__Coriobacteriales;f__uncultured;g__uncultured | 3 | 4 | 4 |
| c__Bacteroidia;o__Bacteroidales;f__Bacteroidales_RF16_group;g__Bacteroidales_RF16_group | 1 | 2 | 3 |
| c__Bacteroidia;o__Bacteroidales;f__Rikenellaceae;g__dgA-11_gut_group | 0 | 0 | 0 |
| c__Bacteroidia;o__Bacteroidales;f__uncultured;g__uncultured | 4 | 4 | 1 |
| c__Campylobacteria;o__Campylobacterales;f__Helicobacteraceae;g__Helicobacter | 0 | 0 | 0 |
| c__Vampirivibrionia;o__Gastranaerophilales;f__Gastranaerophilales;g__Gastranaerophilales | 0 | 0 | 0 |
| c__Deferribacteres;o__Deferribacterales;f__Deferribacteraceae;g__Mucispirillum | 3 | 2 | 2 |
| c__Desulfuromonadia;o__Bradymonadales;f__Bradymonadales;g__Bradymonadales | 0 | 0 | 1 |
| c__Fibrobacteria;o__Fibrobacterales;f__Fibrobacteraceae;g__Fibrobacter | 1 | 0 | 2 |
| c__Bacilli;o__Erysipelotrichales;f__Erysipelatoclostridiaceae;g__UCG-004 | 1 | 0 | 0 |
| c__Bacilli;o__Erysipelotrichales;f__Erysipelotrichaceae;g__Catenisphaera | 0 | 0 | 2 |
| c__Bacilli;o__Erysipelotrichales;f__Erysipelotrichaceae;g__Erysipelotrichaceae_UCG-006 | 4 | 1 | 1 |
| c__Bacilli;o__Erysipelotrichales;f__Erysipelotrichaceae;g__Erysipelotrichaceae_UCG-009 | 5 | 5 | 5 |
| c__Bacilli;o__Erysipelotrichales;f__Erysipelotrichaceae;g__Solobacterium | 2 | 1 | 1 |
| c__Bacilli;o__RF39;f__RF39;g__RF39 | 1 | 0 | 0 |
| c__Clostridia;__;__;__ | 1 | 1 | 2 |
| c__Clostridia;o__Clostridiales;f__Clostridiaceae;g__Clostridium_sensu_stricto_6 | 1 | 2 | 1 |
| c__Clostridia;o__Eubacteriales;f__Eubacteriaceae;g__Pseudoramibacter | 5 | 5 | 4 |
| c__Clostridia;o__Lachnospirales;f__Lachnospiraceae;g__Agathobacter | 1 | 0 | 1 |
| c__Clostridia;o__Lachnospirales;f__Lachnospiraceae;g__Anaerostipes | 5 | 5 | 4 |
| c__Clostridia;o__Lachnospirales;f__Lachnospiraceae;g__Butyrivibrio | 5 | 5 | 5 |
| c__Clostridia;o__Lachnospirales;f__Lachnospiraceae;g__Dorea | 0 | 3 | 1 |
| c__Clostridia;o__Lachnospirales;f__Lachnospiraceae;g__Fusicatenibacter | 3 | 1 | 3 |
| c__Clostridia;o__Lachnospirales;f__Lachnospiraceae;g__Howardella | 1 | 2 | 2 |
| c__Clostridia;o__Lachnospirales;f__Lachnospiraceae;g__Lachnospira | 1 | 0 | 0 |
| c__Clostridia;o__Lachnospirales;f__Lachnospiraceae;g__Lachnospiraceae_AC2044_group | 2 | 2 | 2 |
| c__Clostridia;o__Lachnospirales;f__Lachnospiraceae;g__Lachnospiraceae_NK3A20_group | 4 | 4 | 4 |
| c__Clostridia;o__Lachnospirales;f__Lachnospiraceae;g__Lachnospiraceae_NK4A136_group | 0 | 0 | 0 |
| c__Clostridia;o__Lachnospirales;f__Lachnospiraceae;g__Lachnospiraceae_NK4B4_group | 4 | 4 | 4 |
| c__Clostridia;o__Lachnospirales;f__Lachnospiraceae;g__Lachnospiraceae_UCG-003 | 5 | 3 | 2 |
| c__Clostridia;o__Lachnospirales;f__Lachnospiraceae;g__Lachnospiraceae_UCG-004 | 1 | 0 | 0 |
| c__Clostridia;o__Lachnospirales;f__Lachnospiraceae;g__Lachnospiraceae_UCG-007 | 3 | 2 | 4 |
| c__Clostridia;o__Lachnospirales;f__Lachnospiraceae;g__Lachnospiraceae_UCG-008 | 4 | 3 | 4 |
| c__Clostridia;o__Lachnospirales;f__Lachnospiraceae;g__Marvinbryantia | 0 | 1 | 1 |
| c__Clostridia;o__Lachnospirales;f__Lachnospiraceae;g__Shuttleworthia | 5 | 5 | 5 |
| c__Clostridia;o__Lachnospirales;f__Lachnospiraceae;g__Syntrophococcus | 5 | 5 | 5 |
| c__Clostridia;o__Lachnospirales;f__Lachnospiraceae;g__[Eubacterium]_eligens_group | 3 | 4 | 4 |
| c__Clostridia;o__Lachnospirales;f__Lachnospiraceae;g__[Eubacterium]_ruminantium_group | 3 | 3 | 4 |
| c__Clostridia;o__Lachnospirales;f__Lachnospiraceae;g__[Eubacterium]_ventriosum_group | 4 | 5 | 4 |
| c__Clostridia;o__Lachnospirales;f__Lachnospiraceae;g__[Eubacterium]_xylanophilum_group | 2 | 3 | 2 |
| c__Clostridia;o__Lachnospirales;f__Lachnospiraceae;g__[Ruminococcus]_gauvreauii_group | 0 | 1 | 2 |
| c__Clostridia;o__Oscillospirales;f__Butyricicoccaceae;g__UCG-008 | 0 | 1 | 1 |
| c__Clostridia;o__Oscillospirales;f__Butyricicoccaceae;g__UCG-009 | 0 | 0 | 0 |
| c__Clostridia;o__Oscillospirales;f__Oscillospiraceae;g__UCG-003 | 0 | 0 | 0 |
| c__Clostridia;o__Oscillospirales;f__Ruminococcaceae;g__CAG-352 | 0 | 2 | 0 |
| c__Clostridia;o__Oscillospirales;f__Ruminococcaceae;g__Negativibacillus | 1 | 2 | 2 |
| c__Clostridia;o__Oscillospirales;f__Ruminococcaceae;g__Paludicola | 5 | 5 | 5 |
| c__Clostridia;o__Peptostreptococcales-Tissierellales;f__Anaerovoracaceae;__ | 4 | 4 | 2 |
| c__Clostridia;o__Peptostreptococcales-Tissierellales;f__Anaerovoracaceae;g__Family_XIII_UCG-001 | 0 | 0 | 0 |
| c__Clostridia;o__Peptostreptococcales-Tissierellales;f__Peptostreptococcaceae;g__Intestinibacter | 0 | 1 | 0 |
| c__Clostridia;o__Peptostreptococcales-Tissierellales;f__Peptostreptococcaceae;g__Romboutsia | 0 | 0 | 0 |
| c__Negativicutes;o__Acidaminococcales;f__Acidaminococcaceae;g__Acidaminococcus | 2 | 1 | 2 |
| c__Negativicutes;o__Veillonellales-Selenomonadales;f__Selenomonadaceae;__ | 1 | 1 | 1 |
| c__Negativicutes;o__Veillonellales-Selenomonadales;f__Selenomonadaceae;g__Mitsuokella | 3 | 3 | 3 |
| c__Negativicutes;o__Veillonellales-Selenomonadales;f__Selenomonadaceae;g__Selenomonas | 4 | 4 | 2 |
| c__Negativicutes;o__Veillonellales-Selenomonadales;f__Selenomonadaceae;g__uncultured | 2 | 2 | 2 |
| c__Negativicutes;o__Veillonellales-Selenomonadales;f__Veillonellaceae;g__Allisonella | 1 | 2 | 1 |
| c__Negativicutes;o__Veillonellales-Selenomonadales;f__Veillonellaceae;g__Dialister | 2 | 3 | 5 |
| c__Alphaproteobacteria;o__Rhodospirillales;f__uncultured;g__uncultured | 1 | 1 | 3 |
| c__Gammaproteobacteria;o__Aeromonadales;f__Succinivibrionaceae;__ | 5 | 5 | 5 |
| c__Gammaproteobacteria;o__Aeromonadales;f__Succinivibrionaceae;g__Succinivibrionaceae_UCG-001 | 5 | 5 | 5 |
| c__Gammaproteobacteria;o__Burkholderiales;f__Oxalobacteraceae;__ | 1 | 0 | 0 |
| c__Gammaproteobacteria;o__Burkholderiales;f__Sutterellaceae;g__Parasutterella | 2 | 5 | 2 |
| c__Kiritimatiellae;o__WCHB1-41;f__WCHB1-41;g__WCHB1-41 | 0 | 0 | 1 |
| c__Lentisphaeria;o__Victivallales;f__Victivallaceae;g__Victivallaceae | 1 | 2 | 2 |
| c__Lentisphaeria;o__Victivallales;f__vadinBE97;g__vadinBE97 | 1 | 1 | 0 |
| c__Bacteroidia;o__Bacteroidales;f__Prevotellaceae;g__uncultured | 1 | 1 | 0 |
| c__Clostridia;o__Christensenellales;f__Christensenellaceae;g__uncultured | 1 | 0 | 0 |
| c__Clostridia;o__Oscillospirales;f__Butyricicoccaceae;g__Butyricicoccus | 1 | 0 | 0 |
| c__Clostridia;o__Peptostreptococcales-Tissierellales;f__Anaerovoracaceae;g__[Eubacterium]_nodatum_group | 1 | 1 | 0 |
| c__Actinobacteria;o__Corynebacteriales;f__Corynebacteriaceae;g__Corynebacterium | 0 | 1 | 0 |
| c__Bacilli;o__Erysipelotrichales;f__Erysipelatoclostridiaceae;g__Catenibacterium | 0 | 2 | 2 |
| c__Clostridia;o__Lachnospirales;f__Lachnospiraceae;g__Lachnospiraceae_ND3007_group | 0 | 1 | 0 |
| c__Clostridia;o__Oscillospirales;f__Oscillospiraceae;g__uncultured | 0 | 1 | 1 |
| c__Clostridia;o__Oscillospirales;f__Ruminococcaceae;g__Faecalibacterium | 0 | 1 | 0 |
| c__Clostridia;o__Peptococcales;f__Peptococcaceae;g__uncultured | 0 | 1 | 0 |
| c__Coriobacteriia;o__Coriobacteriales;f__Coriobacteriaceae;g__Collinsella | 0 | 0 | 1 |
| c__Coriobacteriia;o__Coriobacteriales;f__Eggerthellaceae;g__Slackia | 0 | 0 | 2 |
| c__Bacilli;o__Erysipelotrichales;f__Erysipelotrichaceae;g__Turicibacter | 0 | 0 | 1 |
| c__Clostridia;o__Oscillospirales;f__Oscillospiraceae;g__Oscillospira | 0 | 0 | 1 |
| c__Clostridia;o__Peptococcales;f__Peptococcaceae;g__Peptococcus | 0 | 0 | 1 |
| c__Gammaproteobacteria;o__Aeromonadales;f__Succinivibrionaceae;g__Succinivibrio | 0 | 0 | 1 |

**
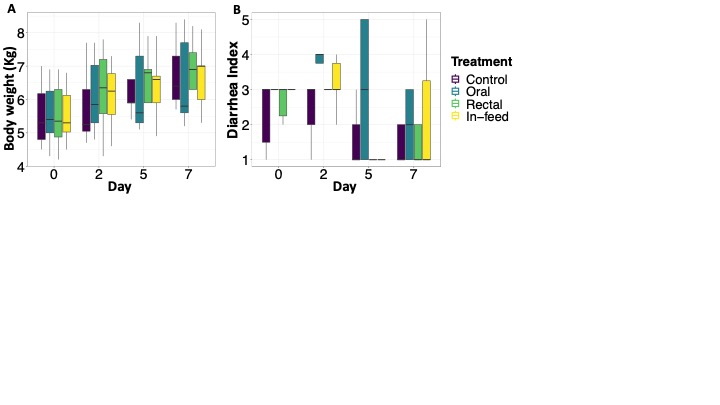
**

**Fig. S1**: Effect of route of delivery of FMT on body weight (A) and Diarrhea Index (B) of pigs.


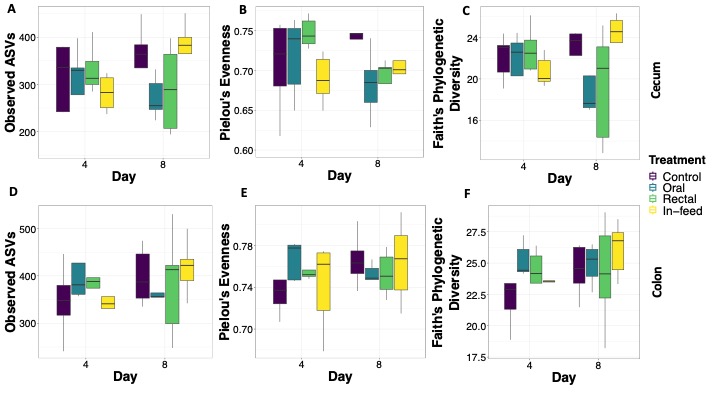


**Fig. S2**: Effects of FMT route of delivery on microbiota dynamics cecum and colon digesta. Alpha diversity of cecum digesta represented by (A) Number of Observed ASVs; (B) Pielou’s Evenness; (C) Faith’s phylogenetic diversity. Alpha diversity of colon digesta represented by (D) Number of Observed ASVs; (E) Pielou’s Evenness; (F) Faith’s phylogenetic diversity.


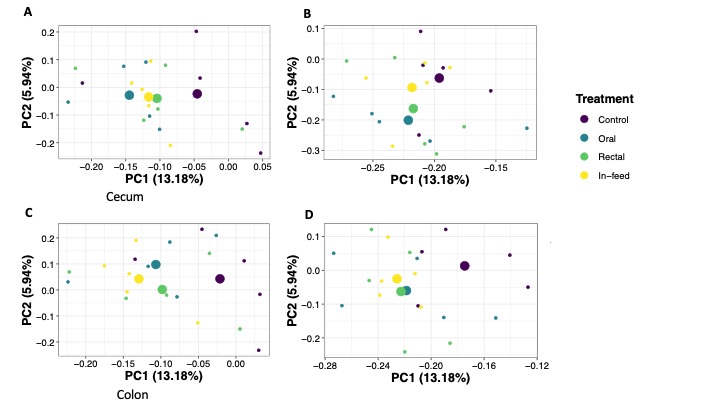


**Fig. S3**: Effects of FMT route of delivery on microbiota dynamics cecum and colon digesta. Beta diversity of cecum digesta represented by Jaccard similarity for cecum on (A) day 4; (B) day 8; and colon on (C) day 4; (D) day 8.


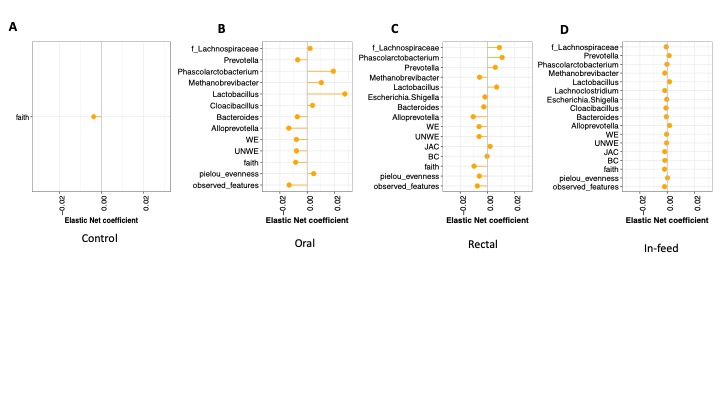


**Fig. S4**: Prediction of the rejection of donor taxa in recipients based on the pre-FMT recipient microbiota features using cross-validated Elastic Net regularized regression. Variable coefficient and directionality of the full model for (A) Control; (B) Oral; (C) Rectal; (D) In-feed.
